# NIR-IIb fluorescence-image guided synergistic surgery/starvation/chemodynamic therapy: an innovative treatment paradigm for malignant non-small cell lung cancers

**DOI:** 10.7150/thno.83753

**Published:** 2023-04-01

**Authors:** Xuejiao Han, Yingtao Zhong, Chao Mi, Zhiguo He, Jingsi Gu, Xiaoyong Dai, Chenguang Ma, Chunyan Feng, Huaqing Chen, Zebin Lan, Zhiyong Guo, Laiqiang Huang, Baozhu Zhang, Bing Guo, Qingwei Meng

**Affiliations:** 1 Department of Medical Oncology, Harbin Medical University Cancer Hospital, Harbin 150081, China. E-mail address: mengqw@hrbmu.edu.cn; 2 School of Science, Shenzhen Key Laboratory of Flexible Printed Electronics Technology, Harbin Institute of Technology, Shenzhen 518055, China. E-mail address: guobing2020@hit.edu.cn; 3 UTS-SUStech Joint Research Centre for Biomedical Materials and Devices, Department of Biomedical Engineering, Southern University of Science and Technology, Shenzhen, Guangdong 518055, China; 4 Education Center and Experiments and Innovations, Harbin Institute of Technology, Shenzhen 518055, China; 5 Institute of Biopharmaceutical and Health Engineering, Shenzhen Key Laboratory of Gene and Antibody Therapy, State Key Laboratory of Chemical Oncogenomics, Shenzhen International Graduate School, Tsinghua University, Shenzhen, Guangdong 518055, China; 6 Department of Radiation Oncology, People's Hospital of Shenzhen Baoan District, The Second Affiliated Hospital of Shenzhen University, Shenzhen, Guangdong 518100, China

**Keywords:** downconversion nanoparticles, NIR-II fluorescence imaging, chemodynamic therapy, early tumors, non-small cell lung cancers

## Abstract

**Background:** Currently, the prognosis and survival rate for patients bearing non-small cell lung cancer (NSCLC) is still quite poor, mainly due to lack of efficient theranostic paradigms to exert in time diagnostics and therapeutics.

**Methods:** Herein, for NSCLC treatment, we offer a customized theranostic paradigm, termed NIR-IIb fluorescence diagnosis and synergistic surgery/starvation/chemodynamic therapeutics, with a newly designed theranostic nanoplatform PEG/MnCuDCNPs@GOx. The nanoplatform is composed of brightly NIR-II emissive downconversion nanoparticles (DCNPs)-core and Mn/Cu-silica shell loaded with glucose oxidase (GOx) to achieve synergistic starvation and chemodynamic therapy (CDT).

**Results:** It is found that 10% Ce^3+^ doped in the core and 100% Yb^3+^ doped in the middle shell greatly improves the NIR-IIb emission up to even 20.3 times as compared to the core-shell DCNPs without Ce^3+^ doping and middle shell. The bright NIR-IIb emission of the nanoplatform contributes to sensitive margin delineation of early-stage NSCLC (diameter < 1 mm) with a signal-to-background ratio (SBR) of 2.18, and further assists in visualizing drug distribution and guiding surgery/starvation/chemodynamic therapy. Notably, the starvation therapy mediated by GOx-driven oxidation reaction efficiently depletes intratumoral glucose, and supplies H_2_O_2_ to boost the CDT mediated by the Mn^2+^ and Cu^2+^, which consequently realized a highly effective synergistic treatment for NSCLC.

**Conclusion:** This research demonstrates an efficient treatment paradigm for NSCLC with NIR-IIb fluorescence diganosis and image-guided synergistic surgery/starvation/chemodynamic therapeutics.

## Introduction

Nowadays, advanced non-small cell lung cancer (NSCLC) is still devastated for human beings, with a poor prognosis 1,2. As the primary subtype of lung cancer, NSCLC accounts for more than 85% of the diagnosed lung cancer and claims millions of lives annually 3. Although tremendous efforts have been devoted to innovating the NSCLC treatment paradigms including surgery, chemo-/targeted and immunotherapy, currently the median survival is still unsatisfactory, ranging from 8 to 36 months for patients 3-8. The main reasons for the low median survival rates include (i) difficulty in highly sensitive detection of tumors in early-stage, which leads to rapid growth, infiltration and metastasis of tumors, (ii) lack of efficient real-time imaging technology to pinpoint tumor margin for precision surgery, (iii) ease in formation of multiple drug resistance for chemo- and targeted therapy, and (iv) limited response rate for immunotherapy and even super progression after initiation of immunotherapy 3-8. Therefore, it is desperate to bring in more efficient theranostic paradigms to circumvent these problems, and importantly achieve dual early diagnosis and in-time effective ablation of tumors.

Sensitive diagnosis of early-stage microscopic tumors (diameter < 1 mm) is still quite challenging for conventional imaging modalities, such as visible/NIR-I optical imaging (vis/NIR-I OI, 400-900 nm), computed tomography (CT), magnetic resonance imaging (MRI), ultrasound imaging (US), positron emission tomography (PET), and single-photon emission computed tomography (SPET) 9-15. It is because they generally exhibit certain drawbacks, such as limited penetration depth (for vis/NIR-I OI), unsatisfactory sensitivity (for US and MRI), low signal-to-background ratio (SBR, for vis/NIR-I OI, US, CT and MRI), and ionizing radiation to patients and physicians (for CT and PET). In contrast, recently we and others have confirmed the NIR-II optical imaging could realize in time* in vivo* imaging with good spatiotemporal resolution, high sensitivity, deep penetration, large SBR and non-invasiveness, which paves the way for sensitive diagnosis of early-stage microscopic tumors 13-15. Notably, the fidelity of NIR-II optical imaging heavily depends on the brightness of the contrast agents 13-15, for which both organic materials (*e.g.*, dyes and conjugated polymers) and inorganic ones (carbon nanotubes, quantum dots, plasm nanoparticles), and lanthanide-doped downconversion nanoparticles (DCNPs) have been developed 16-20. Among these materials, DCNPs have attracted increasing attention in application of monitoring physiological and pathological processes 16-26. The primary reason is that they could be customized to offer nonphotobleached emission in the transparent NIR-IIb window (1500-1700 nm) with much lower background interference, suppressed scattering and deeper penetration as compared to the vis/NIR-I and even NIR-IIa window (1000-1300 nm) *via* adjusting their composition and morphology 21-24. In addition, these DCNPs could be decorated with therapeutic functions *via* surface modification and/or co-loading of drugs 24-31. Therefore, it is envisioned that highly bright NIR-II DCNPs would be good theranostic candidates for sensitive imaging of early-stage microscopic NSCLC tumors, and as well as NSCLC therapy, although the related research is quite rare so far.

X-dynamic therapies such as photodynamic therapy (PDT) and chemodynamic therapy (CDT), emerging as precision alternatives to conventional NSCLC therapies (*e.g.*, surgery, chemo-/targeted and immuno- therapy), present superiority in minimal invasiveness and tumor specificity 30-35. Importantly, X-dynamic therapies can locally ablate tumors with destructive reactive oxygen species (ROS) and avoid multiple drug resistance. Different from normal tissues, tumor microenvironment (TME) shows mild acidity, hypoxia, low catalase and overproduced hydrogen peroxide (H_2_O_2_) and high concentration of glutathione (GSH) which is an active antioxidant to consume intracellular ROS 33. However, PDT hardly takes efficacy on either hypoxic, or GSH-rich TME, or deep tumors due to its oxygen-dependent nature, the ROS consumption by intratumoral GSH, and limited penetration depth of the external irradiation light, respectively 32,33. Moreover, administration of photosensitizers could make patients susceptible to sunlight cytotoxicity 32. Instead of being compromised, CDT is exclusively activated in TME, and this contributes to wiping out such concerns on side-effects, tumoral depth and oxygen level 33-37. It is because CDT exert tumor ablation with highly virulent hydroxyl radicals (•OH) which is generated in Fenton-like reaction between CDT reactants including metal ions (*e.g.*, Fe^2+^, Co^2+^, Mn^2+^, Cu^+^) and H_2_O_2_ under acid conditions, which exist in tumors rather than normal tissues 33-36. Notably, the CDT efficacy is highly dependent on the Fenton-like reaction conditions including H_2_O_2_ concentration, acidity and metal ion species 27,28,38-41. Since tumors generally exhibit insufficient H_2_O_2_ level (≈10×10^-6^ M), rich-GSH and mild acidity, actions including introduction of continuous H_2_O_2_ supply and GSH depletion, and selection of highly reactive metal ion species in mild acidic tumors, contribute to highly efficient CDT 27,28,38-42, and importantly would offer excellent treatment outcomes on NSCLC, which has rarely been investigated so far.

**Scheme 1:**
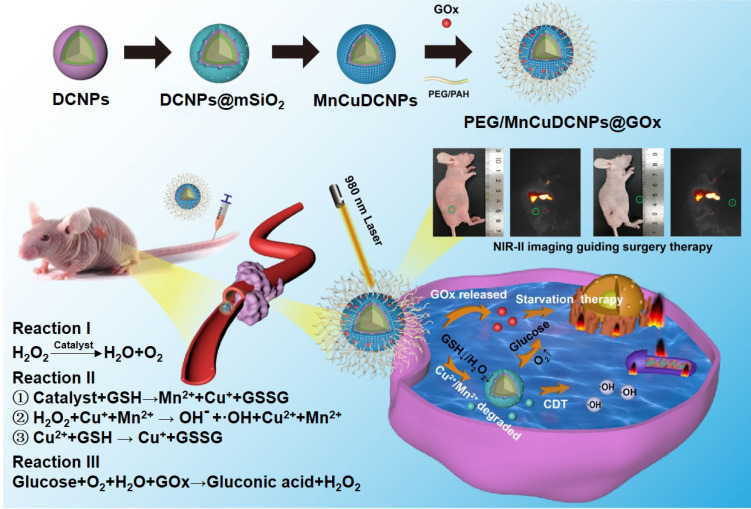
The illustration for theranostic nanoplatform for efficient NIR-IIb fluorescence diagnosis and synergistic surgery/starvation/chemodynamic therapeutics of non-small lung cancers in early stages.

Previously, we and others have demonstrated that lanthanide-doped nanoparticles (NPs) can readily integrate “all-in-one” theranostic platforms for image-guided X-dynamic/combinatory therapies with real-time monitoring of the pharmaceutic kinetics and distribution. In this contribution, we formulated multiple functional DCNP nanoplatform to innovate NSCLC treatment paradigm with dual diagnosis of early-stage NSCLC and image-guided synergistic surgery/starvation/chemodynamic therapy. We designed and synthesized PEGylated and glucose oxidase (GOx) loaded DCNPs (PEG/ MnCuDCNPs @GOx), which is composed of the bright NIR-II emissive DCNP core and the Mn/Cu-silica shell, for sensitive diagnosis of early NSCLC, and image-guided synergistic surgery/starvation/chemodynamic therapy (Scheme 1). The rationales for the theranostic nanoplatform design are as follows. First, through carefully tuning element composition of DCNP core (β-NaYF_4_:Yb/Er/Ce@NaYbF_4_@NaYF_4_), a highly bright NIR-II emissive DCNP core is produced, offering highly sensitive detection and image-guided surgery of NSCLC submillimeter tumors in early stage 21-26. Second, GOx can catalyze glucose into gluconic acid and H_2_O_2_, which not only enhances CDT, but also chock off the energy supply, offering combinatory therapeutics of starvation therapy and CDT, and thus greatly boosts the ultimate anticancer treatment outcomes 39,40,43. Third, copper (Cu) shell offers Cu^2+^ to deplete GSH with production of Cu^+^ which further catalyze H_2_O_2_ to yield •OH 40,41. Importantly, the Cu^+^-catalysis occurs in both weakly acidic and neutral conditions with a Fenton-like reaction rate ten-fold higher than that of the most common CDT agent Fe^2+^ 40. Forth, Mn shell provides Mn^2+^ as Fenton-like catalyst reacts with H_2_O_2_ with effectively *in situ* generation of •OH for CDT and O_2_ for subsequent catalytic starvation reaction 44-46. Fifth, manganese /copper (Mn^2+^Cu^2+^) silicate shell acts as gate-keeper to minimize side effects and its decomposition leads to the release of GOx, triggering the generation of O_2_ and •OH. For the synthesized nanoplatform, we systematically evaluated the photophysical properties, the bioimaging and therapeutic performance on NSCLC tumors *in vitro* and *in vivo*.

## Results and Discussion

### Design and Synthesis of β-NaYF_4_:Er,Ce,Yb@NaYbF_4_@NaYF_4_

**Figure 1:**
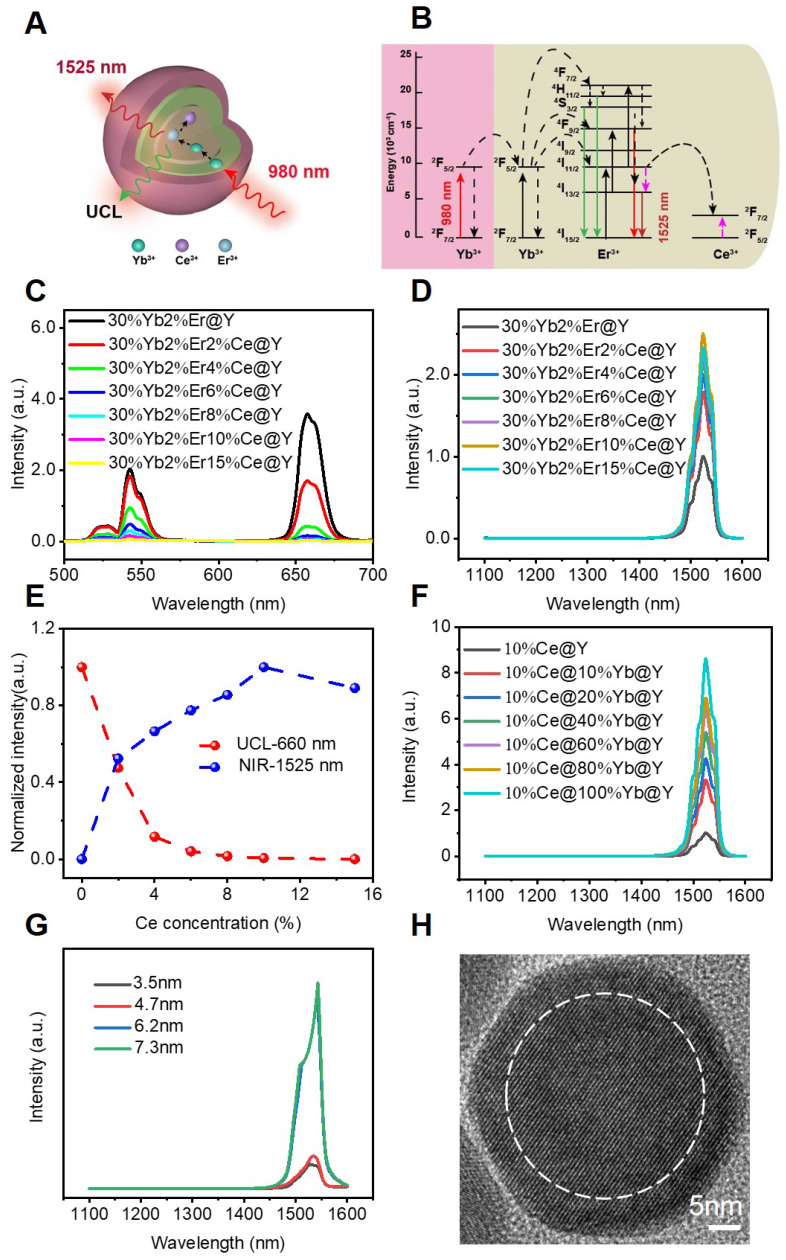
** (A)** Schematic illustration for the proposed mechanisms of energy-transfer in DCNPs (NaYF_4_:Er,Ce,Yb@NaYbF_4_@NaYF_4_). **(B)** The energy level diagrams of UC and DC process of the Ce^3+^, Er^3+^, and Yb^3+^ in designed DCNPs. **(C)** UC and **(D)** DC emission spectra, **(E)** corresponding integral intensity upon 980 nm excitation. **(F)** DC luminescence spectra of the DCNPs with 0 to 100% Yb^3+^ doping. **(G)** DC luminescence spectra of the DCNPs with different NaYF_4_ shell thicknesses. **(H)** HRTEM image of the as-prepared core-shell-shell DCNPs.

The core-shell-shell structured DCNPs with bright NIR-IIb emission were designed and synthesized* via* customizing the element contents to tune the upconversion (UC) and downconversion (DC) processes. The DCNPs were composed of a Ce^3+^, Er^3+^and Yb^3+^ co-doped NaYF_4_ core coated by a NaYbF_4_ middle shell and a NaYF_4_ passive shell (Figure 1A). The Yb^3+^ sensitizers were integrated into the nanosystem to assist in producing 1525 nm luminescence. Firstly, the Yb^3+^ ions in the middle shell would harvest 980 nm laser photons and then were excited from ^2^F_7/2_ to ^2^F_5/2_. The energy was then transferred from Yb^3+^ in the middle layer to the Yb^3+^ in the core of NaYF_4_:Er,Ce,Yb@NaYbF_4_@NaYF_4_. Subsequently, an efficient energy transfer from Yb^3+^ to Er^3+^ would excite the Er^3+^ to the intermediate energy level of^ 4^I_11/2_, which had a short-lived lifetime and is beneficial for the following energy transfer. The excited Er^3+^ of ^4^I_11/2_ state would relax non-radiatively to the ^4^I_13/2_ level and then radiatively to the ^4^I1_5/2_ level to produce the 1525 nm DC emission. Furthermore, there was also energy transfer^ 4^F_9/2_ →^4^I_15/2_ and ^2^H_11/2_/^4^S_3/2_ →^ 4^I_15/2_ of Er^3+^, which brought in the UC emission of 640-680 nm and 510-560 nm, respectively. Specifically, the 1525 nm DC emission originated from the rapid non-radiative decay from the intermediate energy state^ 4^I_11/2_ into the ^4^I_13/2_ level could contribute to the NIR-IIb bioimaging *in vitro* and *in vivo* (Figure 1B).

Notably, there exist two competing processes for the DC luminescence. The first is the well-known UC emission process through simultaneous two-photon absorption that excites the ^4^I_11/2_ level to higher levels for subsequent UC emission. The second is the quenching of the excited ^4^I_13/2_ state caused by the OH^-^ group when the DCNPs are transferred to an aqueous solution. Recent studies have demonstrated that Ce^3+^ ions could significantly decrease the lifetime of intermediate ^4^I_11/2_ energy state of Er^3+^ and thus were found to suppress the UC pathway of the first competing process and enhance DC process for improved NIR-IIb under 980 nm laser irradiation 17, 21. Therefore, we carefully adjusted the doping concentration of Ce^3+^ from 0% to 15% in the core of NaYF_4_:Er,Ce,Yb@NaYbF_4_@NaYF_4_ to seek the optimal dopant concentration for the brightest emission in 1525 nm. Surprisingly, when increasing the Ce^3+^ content, the UC intensity of the NPs dropped sharply (Figure 1C), while the NIR-IIb emission intensity increased significantly, reaching to a plateau at 10 wt% of Ce^3+^ doping. The maximal NIR-IIb emission of the NPs with 10 wt% of Ce^3+^ doping was nearly 2.5-fold higher than that of the NPs without Ce^3+^ doping (Figure 1D). This suggests that the Ce^3+^ doping successfully enriched population of ^4^I_13/2_ energy state of Er^3+^ in the DCNP core, which can intensify the 1525 nm fluorescence (Figure 1E). Considering these results, 10 wt% of Ce^3+^ doping was chosen as the optimized condition for the DCNP core formulation.

To further increase the DC emission and improve the NIR-IIb brightness, we firstly try to choose Yb^3+^ doped in the middle shell to synthesize a core-shell-shell DCNPs. The Yb^3+^ ions are extensively used as sensitizer ions and have been shown to function as an energy-harvesting center which could capture excitation photons 47. It is noticed that when increasing the content of Yb^3+^ in the middle shell from 0% to 100%, the 1525 nm emission increased even 8.5 times, indicating that Yb^3+^ doping in the middle shell contributes to an significant enhancement of NIR-IIb brightness (Figure 1F). For one thing, implanting Yb^3+^ ions into the middle shell of DCNPs could increase the distance between Er^3+^ ions and OH^-^ group when the DCNPs are transferred to aqueous solutions, attenuating the quenching effect. For another thing, the doped Yb^3+^ ions can enable pump energy to Er^3+^ activators, alleviating the possible energy migration. By virtue of such a doped core-shell-shell design, the DCNPs achieved a 20.3-fold increase in the DC emission at 1525 nm compared with the pristine core-shell structured NPs (NaYF_4_:2%Er,30%Yb@NaYF_4_). Similar dopent modulating strategies have been applied to enhance the DC emission at NIR-II window as well by optimizing the Ce^3+^ concentration in DCNPs cores, achieved a 9-fold and 12.3-fold increase at 1550 nm for Er-RENPs (denoted as NaYbF_4_:2%Er,2%Ce@NaYF_4_) 21 and at 1525 nm for NaYF_4_:Lu,Yb,Er@NaGdF_4_:Yb,Ce^3+^ 28, respectively. Our design not only optimized the Ce^3+^ concentration in the DCNPs core, but also highlighted the use of Yb^3+^-doped middle shell, combination of which has realized an substantial increase of 20.3 folds in the NIR-IIb emission. The bright NIR-IIb fluorescence should be highly beneficial for improving the *in vivo* imaging fidelity due to the much reduced tissue scattering and interference 22. Finally, a passive NaYF_4_ coating was applied at the outermost layer, which could minimize the quenching effects of surface defects and surrounding solvents when the DCNPs are dispersed in water. Apart from the element doping, the thickness of outermost shell layer also affects luminescence brightness 48. Christian Würth *et al.* revealed that an outer shell with a thickness of around 5 nm represented the best compromise between efficient UC and bright NIR-II DC emission by studying the quantum yields of the downshifted Er^3+^ emission around 1520 nm 49, so we modulated the NaYF_4_ shell thickness around 5 nm to investigate its protection effect on the NIR-IIb luminescence in this nanosystem. By adding different amount of the shell precusor sequencially into the core-shell dispersion, a passivation shell with a thickness of 3.5 to 7.5 nm was grown around the core-shell seed. When the thicknesses of NaYF_4_ shell varied from 3.5 nm, 4.7 nm to 6.2 nm (Figure S1), the intensity of the 1525 nm luminescence significantly increased, but further increasing the thickness to the 7.3 nm afforded negligible enhancement (Figure 1G), which suggests that 6.2 nm is thick enough to suppress of the quenching effect of surface defects and solvents. Therefore, the 6.2 nm NaYF_4_ shell was chosen as the optimal thickness for the standard material formulation. The high-resolution TEM (HRTEM) result showed the average shell thickness was *c.a.* 6.2 nm (Figure 1H and Figure S3C). Taken together, the β-NaYF_4_:Er,Ce,Yb@NaYbF_4_@NaYF_4_ DCNPs with 10 wt% Ce^3+^ in the core, 100 wt% Yb^3+^ in middle shell and a 6.2 nm-thick outermost NaYF_4_ shell showed the maximal brightness at 1525 nm, which is therefore selected as the basic platform for subsequent experiments.

### Preparation and Characterization of PEG/MnCuDCNPs@GOx

**Figure 2:**
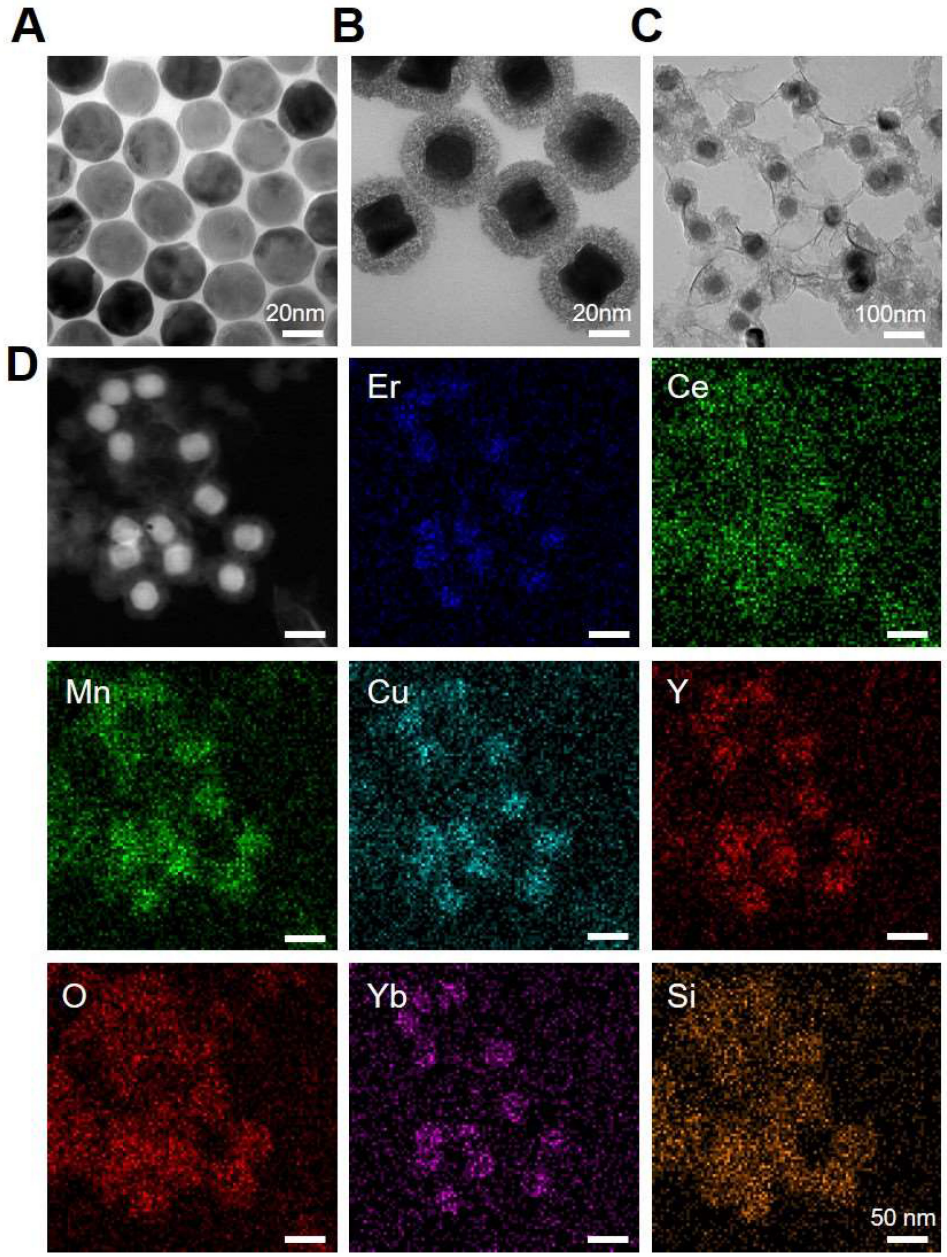
TEM images of** (A)** DCNPs, **(B)** DCNPs@mSiO_2_ and **(C)** PEG/MnCuDCNPs@GOx. **(D)** Element mapping images of PEG/MnCuDCNPs@GOx.

To further customize chemodynamic and starvation therapeutic functions onto the nanoplatform, mesoporous silica (mSiO_2_) shells were firstly coated on the surface of DCNPs by a hydrothermal method. Then the obtained DCNPs@mSiO_2_ further loaded Mn^2+^/Cu^2+^ and GOx to form the MnCuDCNPs@GOx by another hydrothermal process and a dipping method, respectively (Scheme 1). The core, core-shell and core-shell-shell of the DCNPs with diameters of *c.a.* 17.6 nm, 21.6 nm and 28.3 nm are all monodispersed (Figure 2A, Figure S2 and S3). Firstly, the successful decoration of mSiO_2_ onto the surface of DCNPs led to the formation of DCNPs@mSiO_2_ with an increased diameter of *c.a.* 50.5 nm (Figure 2B and Figure S3D). Secondly, during the Mn^2+^Cu^2+^ loading hydrothermal process, copper/manganese silicate was generated by the reaction between the Mn^2+^Cu^2+^ and silicate ions released from DCNPs@mSiO_2_ in the alkaline environment 50-52. The Cu^2+^/Mn^2+^- ammonium complex ions Cu(NH_3_)_4_^2+^ and Mn(NH_3_)_4_^2+^ were persistently generated on the mSiO_2_ template surface until the mSiO_2_ shell were completely decomposed, eventually forming MnCuDCNPs. The loading of the Mn^2+^and Cu^2+^ further increased the diameter of obtained MnCuDCNPs to *c.a.* 71.4 nm (Figure S2C and S3E). Thirdly, GOx can absorb onto the cationic Cu^2+^ and Mn^2+^ ions and concentrate abundant ions to increase the local supersaturation since the surface of GOx at neutral solution is negatively charged 53-54. Finally, to apply the nanoplatform *in vitro* and *in vivo*, PEGylation of MnCuDCNPs@GOx was realized *via* a poly (allylamine hydrochloride) (PAH)-assisted layer-by-layer method to offer water dispersity. This was processed by condensation reaction between -COOH group of PEG and -NH_2_ group on the PAH backbones, which can also enhance the accumulation of the PEG/MnCuDCNPs@GOx at the tumor site *via* enhanced permeability and retention effect 55. The particle size of PEG/MnCuDCNPs@GOx was increased to *c.a.* 77.5 nm as verified by TEM and DLS (Figure 2C and Figure S3F). Energy-dispersive spectroscopy (EDS) (Figure S4) proves the elemental composition and the elements of Ce, Er, Yb, Y, Si, O, Cu and Mn were uniformly distributed over the PEG/MnCuDCNPs@GOx (Figure 2D). The zeta potential of samples is shown in Figure S5.

**Figure 3:**
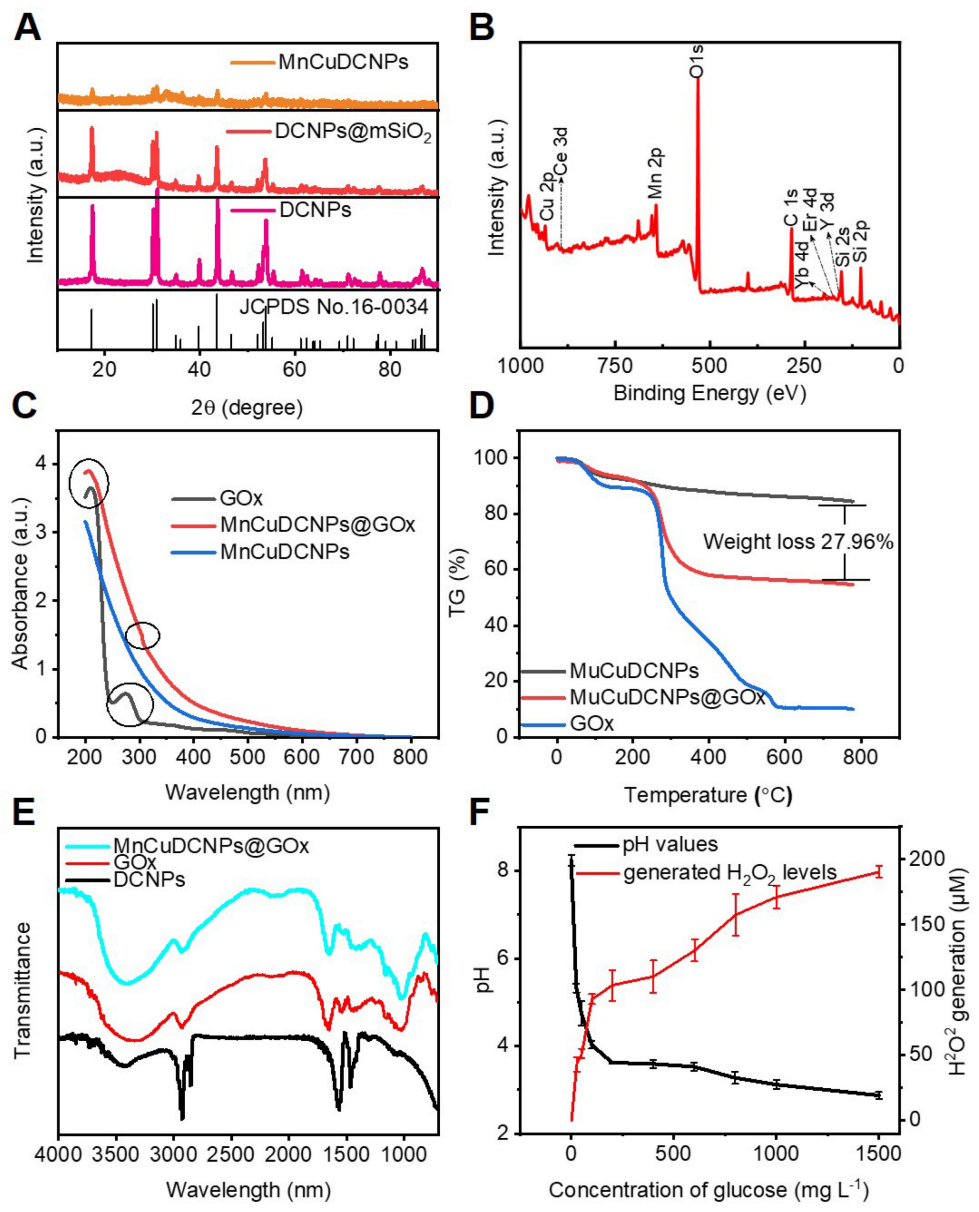
** (A)** XRD patterns of MnCuDCNPs, DCNPs@mSiO_2_ and DCNPs.** (B)** XPS spectra of MnCuDCNPs@GOx: survey spectrum. **(C)** UV-vis spectra of GOx, MnCuDCNPs@GOx, and MnCuDCNPs. ** (D)** TG analysis of GOx, MnCuDCNPs@GOx, and MnCuDCNPs. **(E)** FT-IR spectra of MnCuDCNPs@GOx, DCNPs@mSiO_2_ and DCNPs. **(F)** Generated H_2_O_2_ levels and pH variation of PEG/MnCuDCNPs@GOx after incubation with different concentrations of glucose.

We further systematically characterized the intermediate and final samples to monitor the formulation process and evaluate the sample properties. The XRD patterns of the DCNPs, DCNPs@mSiO_2_, and MnCuDCNPs are shown in Figure 3A. The characteristic diffraction peaks of DCNPs and DCNPs@mSiO_2_ are consistent with the β-NaYF_4_ (JCPDS no.16-0334). Besides, an obvious diffraction peak at 22° shown in DCNPs@mSiO_2_ and the additional characteristic peaks of MnCuDCNPs confirmed the presence of amorphous silica, the copper silicate and manganese silicate (CuMn_6_SiO_12_), respectively 56. As shown in Figure 3B, X-ray photoelectron spectroscopy (XPS) further verified the presence of all the elements of MnCuDCNPs@GOx. Notably, the XPS spectrum of Cu 2p confirmed the existence of Cu^2+^ (Figure S6A), while the spectrum Mn 2p3/2 indicated the presence of Mn^2+^ (641 eV), Mn^3+^ (642 eV), and Mn^4+^ (644 eV) (Figure S6B). Above results indicate the successful coating of the mesoporous silica and the loading of Mn^2+^Cu^2+^ on to the DCNPs.

Figure S7A and B shows the N_2_ adsorption/desorption isotherms and pore-size distribution diagram of MnCuDCNPs. The corresponding Brunauer-Emmett-Teller (BET) surface area was calculated to be 314.68 m^2^g^-1^, the pore volume is around 0.54 cm^3^g^-1^, and the average pore size is nearly 5.01 nm, respectively, which indicate the mesoporous nature of the silica layer. The surface area and mesoporous structure is large enough for substance transfer and active molecular loading 57,58. The corresponding values were decreased to 100.71 m^2^g^-1^, 0.19 cm^3^g^-1^, 4.33 nm after the coating of GOx (Figure S7C and D), respectively. This implies the successful absorption of GOx into the mesoporous silica.

The absorption spectra of GOx, MnCuDCNPs and MnCuDCNPs@GOx were conducted to further confirm the successful loading of GOx. The GOx presented a special peak at 277 nm corresponding to the oxidized flavin group 56. This peak was also retained in the MnCuDCNPs@GOx (Figure 3C). The thermogravimetric analysis (TGA) curves of MnCuDCNPs and MnCuDCNPs@GOx in the range of 100-800 °C indicates that loading ratio of the GOx was 27.96 wt% (Figure 3D). These results demonstrate that GOx was efficiently loaded to form MnCuDCNPs@GOx. The Fourier-transform infrared spectroscopy (FT-IR) spectra was further performed to investigate the surface composition of the prepared MnCuDCNPs@GOx. Figure 3E shows that the DCNPs exhibit the special absorption peaks at 2927, 2855, 1562, and 1466 cm^-1^, which is corresponding to the -CH_2_ and -COOH of oleic acid (OA). The MnCuDCNPs@GOx shows additional obvious peaks at 1082, 950, and 806 cm^-1^, belonging to the stretching vibration bands of Si-OH and Si-O-Si on the coated mSiO_2_ shell 57. Importantly, the characteristic peaks at 1656 cm^-1^ which corresponds to the C=O vibration in GOx are discovered on the spectra of MnCuDCNPs@GOx, indicating the formation of MnCuDCNPs@GOx composites 40.

As GOx can catalyze glucose to produce H_2_O_2_ and gluconic acid 38, we examined the enzyme activity of the loaded GOx by investigating the H_2_O_2_ generation and pH change. Figure 3F shows that more H_2_O_2_ were produced and the pH value decreased when the sample was incubated with higher concentration of glucose. These results confirm that the high enzymatic activity of GOx was hardly affected after being loaded onto the mesoporous silica shell. To investigate stability of the interaction between the absorbed GOx and the MnCuDCNPs, we used the bicinchoninic acid (BCA) kit to colorimetricly detect and quantify the desorbed protein concentration. As shown in the Figure S8A, little protein was detected in the supernatant of the aqueous solution (pH=7.4) for 7 days, and the activity of GOx exhibited negligible decrease in the nanosystem even after 7-day storage at ambient temperature (Figure S8B).

### •OH generation of PEG/MnCuDCNPs@GOx nanoplatform

**Figure 4:**
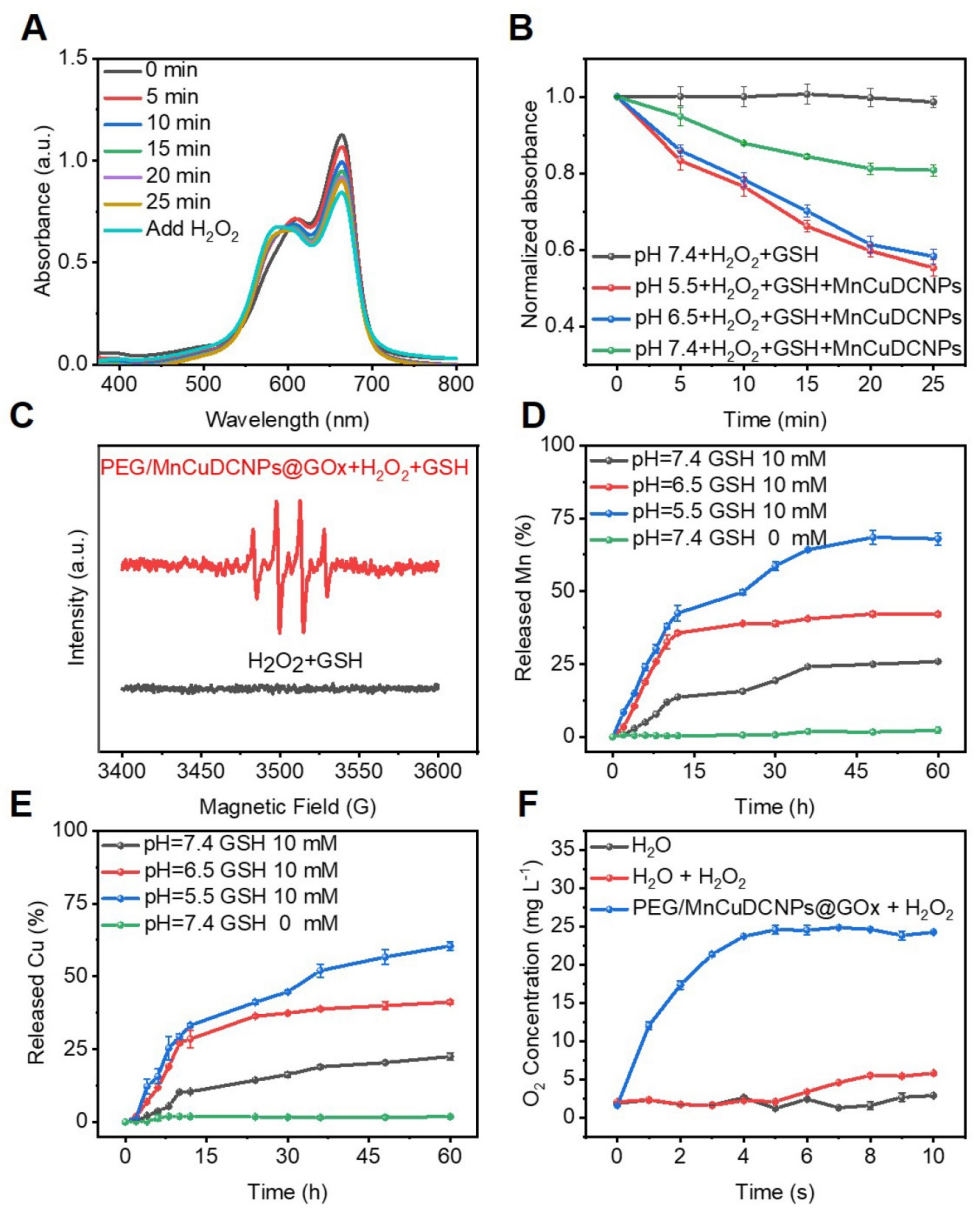
** (A)** The decay curve of MB absorbance in pH=7.4. **(B)** MB absorbance curve variation by GSH-treated MnCuDCNPs and H_2_O_2_ in different pH solutions. **(C)** ESR spectra of •OH detected in different environment. Accumulated releasing profiles of** (D)** Cu^2+^ and **(E)** Mn^2+^ ion from PEG/MnCuDCNPs@GOx with different treatments. **(F)** O_2_ generation test through catalysis of H_2_O_2_ by PEG/MnCuDCNPs@GOx.

To investigate the chemodynamic effect of the PEG/MnCuDCNPs@GOx, the catalytic capability was firstly verified by examining its MB decomposing capability. We used this method to evaluate the •OH production since the MB could be degraded by the •OH produced by the PEG/MnCuDCNPs@GOx mediated Fenton-like reactions. Different from the traditional method using bicarbonate 28,56, we compared MB degradation by adjusting and setting reaction solutions with different pH values (including the pH value of microenvironment for most cancer cells), which was helpful to simulate the internal environment of tumor cells and seek the optimal pH conditions for chemodynamic reaction. As shown in Figure 4A, with the passage of reaction time, the UV absorption intensity of MB decreases continuously when incubated with MnCuDCNPs, GSH and H_2_O_2_ at pH 7.4, implying the generation of •OH. After 25 min, the re-addition of H_2_O_2_ into the system leads to the continuous decline of the intensity, indicative of the dependence of •OH generation on H_2_O_2_ concentration. When the pH decreases from 7.4 to 5.5, the MB degradation efficiency was obviously elevated due to higher production of •OH and ultimately led to a 50% MB degradation (Figure 4A and S9), suggesting that the degradation of MB is more significant when incubated in weakly acidic environment (Figure 4B). These results demonstrate that the nanosystem can effectively catalyze H_2_O_2_ decomposition by the Mn^2+^Cu^2+^- mediated Fenton-like reaction and produce •OH, and the efficacy is highly dependent on H_2_O_2_ concentration and solution acidity. To further confirm the production of •OH, we used 5,5-dimethyl-1-pyrroline-N-oxide (DMPO) to capture free radicals, as the short-lived free radicals could be generated to comparative long-lived radical-DMPO adducts. ESR spectrum shows that the addition of PEG/MnCuDCNPs@GOx into H_2_O_2_ and GSH aqueous solution induced the production of plenty of •OH (Figure 4C). These results suggest that the PEG/MnCuDCNPs@GOx could be activated for CDT in tumor microenvironment. Considering that GOx-triggered glucose oxidation can effectively produce abundant H_2_O_2_ and the •OH production is dependent on H_2_O_2_ concentration (Figure 3F), our rationality of boosting CDT efficacy by adding GOx is thus also further confirmed.

Since metal ions have to be fully released from the nanosystem to react with the free H_2_O_2_ and GSH in the TME, to effectively mediate Fenton-like reactions, we tested the released Mn^2+^ and Cu^2+^ concentration by ICP-OES tests under different pH conditions to evaluate the availability of them in the weakly acid TME. Figure 4D and E shows that the GSH efficaciously initialized the degradation of PEG/MnCuDCNPs@GOx, releasing Mn and Cu ions persistently into the solution. When the pH of the solution decreased from 7.4 to 5.5, the Mn and Cu ion releasing was accelerated. This indicates Cu and Mn ions can be well released from the nanosystem to aid the •OH production in acid TME. In addition to the capability of producing •OH *in situ* for CDT, Mn^2+^Cu^2+^ were also reported to be capable of catalyzing H_2_O_2_ decomposition for O_2_ production (Reaction I) 46. Therefore, we also tested the real-time O_2_ generation to monitor the decomposition of H_2_O_2_ by PEG/MnCuDCNPs@GOx. As expected, the O_2_ production was obviously increased after addition of PEG/MnCuDCNPs@GOx into H_2_O_2_ solution (Figure 4F). The result indicates that PEG/MnCuDCNPs@GOx could accelerate H_2_O_2_ decomposition to generate O_2_, which would contribute to the efficient CDT and starvation therapy *in vitro* and* in vivo*. Above results undoubtedly demonstrate that the PEG/MnCuDCNPs@GOx nanoplatform holds great potential for CDT and starvation combinatory therapeutics.

### Cytotoxicity Experiment

Before testing the cytotoxicity, we conducted the fluorescein isothiocyanate (FITC)-labeled cell imaging experiment and intracellular ROS level assay to investigate the cellular uptake of PEG/MnCuDCNPs@GOx and intracellular •OH production, respectively. The A549 lung tumor cells exhibit the clearly green fluorescence after co-incubation with FITC-labeled PEG/MnCuDCNPs@GOx for 2 h, indicating the efficient intracellular uptake of PEG/MnCuDCNPs@GOx (Figure S10). The 2′,7′-dichlorodihydrofluorescein diacetate (DCFH-DA) was used to probe •OH production in A549 cancer cells treated with PEG/MnCuDCNPs@GOx, which can be oxidized by ROS into brightly green fluorescent 2′,7′-dichlorofuorescin (DCF) 46. Figure S11 shows that the cells treated with PEG/MnCuDCNPs@GOx display the strongest green fluorescence, indicating a strong •OH production, while weaker fluorescence is shown in the MnCuDCNPs group and negligible fluorescence was detected in other groups. This is because the GOx catalyzed glucose depletion in A549 cells and produced H_2_O_2_ to promote Mn^2+^Cu^2+^-mediated CDT process.

**Figure 5:**
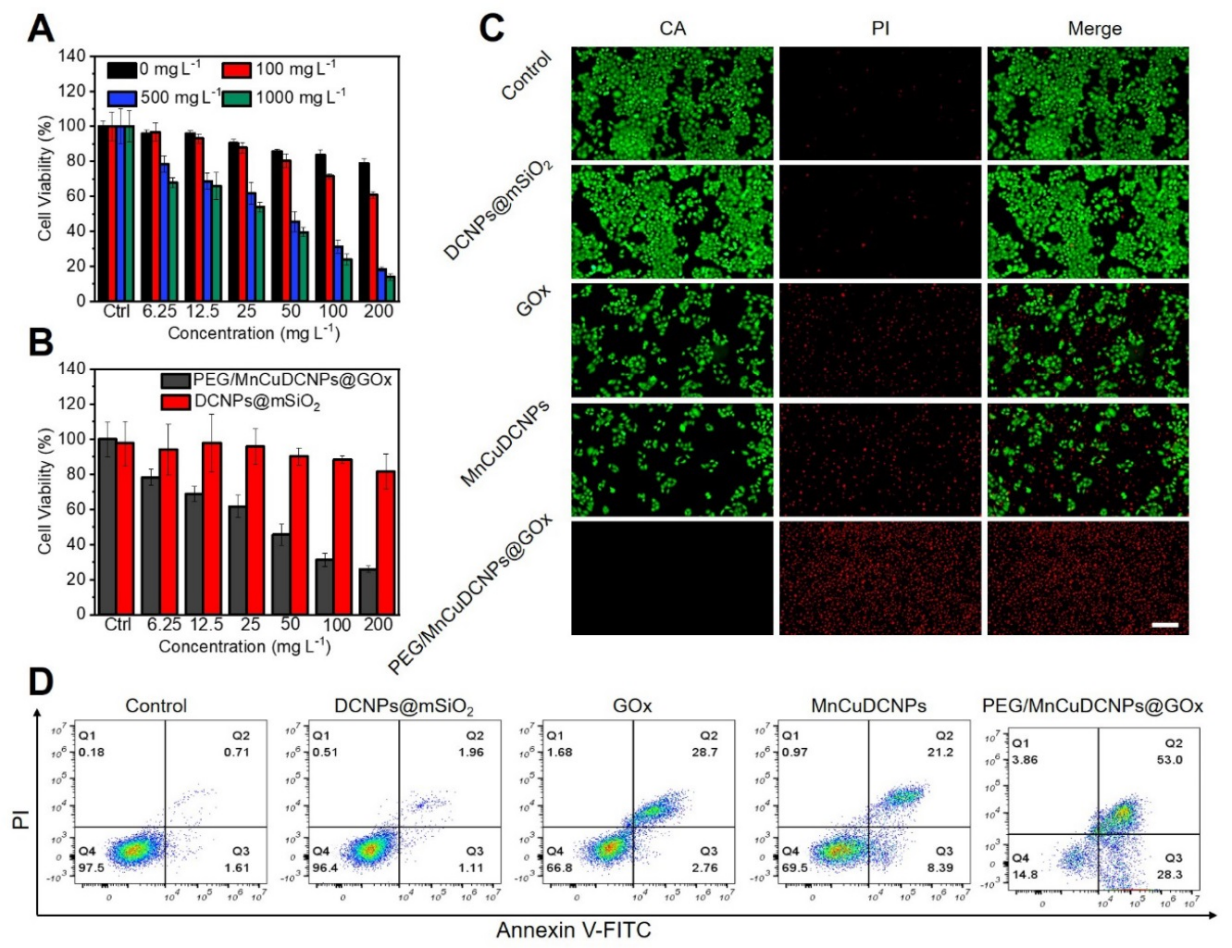
** (A)** A549 cell viabilities treated with PEG/MnCuDCNPs@GOx in DMEM media containing different concentrations of glucose. **(B)** The cell viabilities of A549 after incubated with DCNPs@mSiO_2_ and PEG/MnCuDCNPs@GOx in DMEM media containing 500 mg L^-1^ glucose. **(C)** Confocal microscope images of CA/PI co-stained A549 cells treated with different samples in DMEM media containing glucose. Scale bar: 100 μm.** (D)** Fluorescein-annexin V and PI staining assays of A549 cells treated with different samples.

Encouraged by the qualitative evaluation of •OH generation in cancer cells treated with PEG/MnCuDCNPs@GOx, we further quantitatively verified the therapeutic efficacy in A549 NSCLC cells by standard CCK-8 assay. Various concentrations (0 - 1000 mg L^-1^) of glucose were added to the DMEM medium to simulate the glucose-supplying tumor environment, and then PEG/MnCuDCNPs@GOx (0 - 200 mg L^-1^) was added and co-incubated with cancer cells for 24 h. Figure 5A shows that as the concentrations of both glucose and PEG/MnCuDCNPs@GOx increase, the cytotoxicity would increase. Especially, with 200 mg L^-1^ of PEG/MnCuDCNPs@GOx and 1000 mg L^-1^ of glucose, the A549 NSCLC cell survival was suppressed to *c.a.* 14%. Moreover, when compared with DCNPs@SiO_2_, PEG/MnCuDCNPs@GOx could exhibit far stronger potency towards A459 NSCLC cells at a fixed glucose concentration of 500 mg L^-1^ (Figure 5B). The live-dead staining experiment was conducted to further verify the cytotoxicity results. As shown in Figure 5C, there is almost no cell damage (which is stained in red) in the control group, and increasing amount of dead cells existed in the MnCuDCNPs and GOx treatment, indicative of the therapeutic efficacy of the Mn^2+^Cu^2+^-mediated CDT and GOx-induced starvation therapy, respectively. Significantly, almost all the cells were killed in the PEG/MnCuDCNPs@GOx treated group. Consistent with the cytotoxicity tests, the results also demonstrated the therapeutic efficacy induced by GOx-mediated starvation therapy, and the boosted Mn^2+^Cu^2+^ mediated CDT efficacy. These results combined could tell us the following information. i) PEG/MnCuDCNPs@GOx could exert CDT to ablate cancer cell due to the Mn^2+^Cu^2+^-mediated Fenton-like reactions in the acid TME, whereas the CDT efficacy alone is unsatisfactory; ii) with both the loading of GOx and addition of proper amount of glucose, the therapeutic efficiency of the sample could be dramatically enhanced. The latter phenomenon could result from two aspects. For one thing, the GOx could deplete intratumoral glucose and O_2_ to kill cancer cells by starvation therapy and produce H_2_O_2_ to boost the CDT efficacy; for another, the PEG/MnCuDCNPs@GOx not only initiates Fenton-like reactions to ablate cancer cells, but also catalyze H_2_O_2_ decomposition, releasing O_2_ to afford sustainable glucose depletion with the aid of GOx. We also used the Glucose detection kit (O-toluidine method) to perform the glucose depletion assay *in vitro*. As the concentration of PEG/MnCuDCNPs@GOx increased, the content of glucose gradually decreased (Figure S8C), which further indicated the glucose depletion by the GOx. Therefore, the PEG/MnCuDCNPs@GOx integrating with both the Mn^2+^Cu^2+^ and GOx could serve as a highly efficient nanotherapeutic platform for A459 NSCLC *in vivo* due to the self-boosted combinatory CDT/starvation therapeutics.

Annexin-VFITC Apoptosis Detection Kit was used to further confirm the above results. It shows that in the control and DCNPs@mSiO_2_ groups, the proportion of normal cells exceeds 90%, and the proportions of early-apoptotic and late-apoptotic cells are all less than 4% (Figure 5D). Furthermore, the proportions of apoptotic cells in the groups of GOx, MnCuDCNPs, and PEG/MnCuDCNPs@GOx are higher than 30%, especially in the PEG/MnCuDCNPs@GOx group where the proportion is as high as 81.3%, indicative of the potent anticancer efficacy of the starvation/CDT combinatory therapeutics in contrast with the starvation and CDT alone.

### Early detection of tumor and NIR-II guided tumor surgery

**Figure 6:**
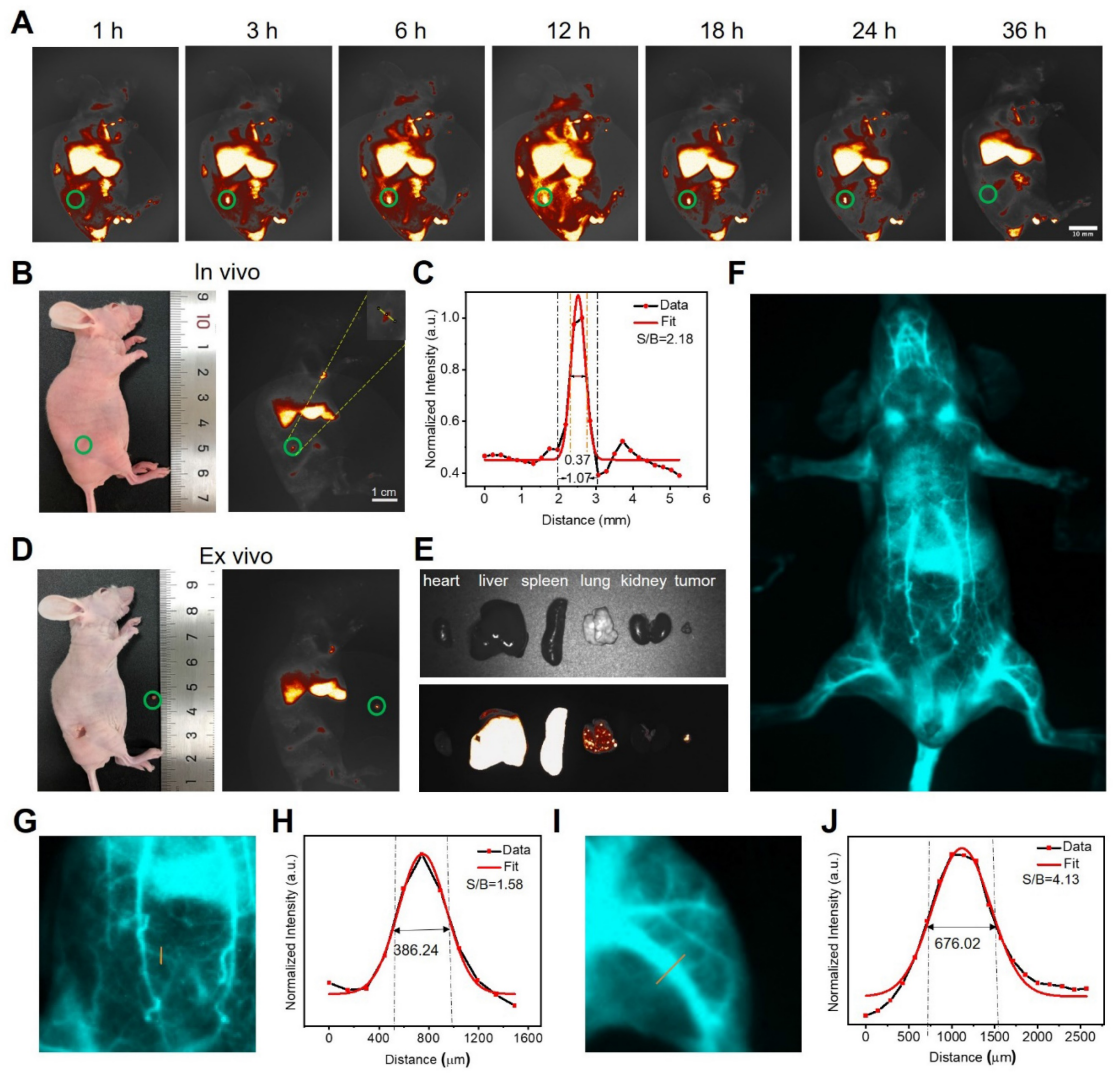
** (A)** Fluorescence images of living mice bearing the subcutaneous xenograft tumor at different time points after tail vein injection of PEG/MnCuDCNPs@GOx (5g L^-1^, 200 µL). **(B)** Fluorescence images of living mice bearing the subcutaneous xenograft tumor with volume of 1 mm^3^. **(C)** Corresonding SBR for fluorescence imaging of early tumor in living mice treated with PEG/MnCuDCNPs@GOx. **(D)** NIR-IIb emission of PEG/MnCuDCNPs@GOx guided early tumor excision and **(E)** *ex vivo* images after animal sacrifice. **(F)** Whole body vascular image in NIR-IIb region. The SBR analysis of NIR-IIb vascular image by plotting the intensity profiles of the cross-sectional (exposure time: 200 ms) corresponding to the abdomen **(G-H)** and leg **(I-J)** vascular, respectively.

As PEG/MnCuDCNPs@GOx has bright NIR-IIb emission, we further explored its potential in tumor diagnosis and image-guided surgery tumor removal* in vivo*. Upon the 980 nm laser excitation, the NIR-IIb fluorescence signal becomes stronger as the PEG/MuCuDCNPs@GOx concentration increased from 0.25 to 4 mg mL^-1^ (Figure S12). Figure S13 shows the photo-stability and long-term bio-stability of PEG/MuCuDCNPs@GOx. The hydrophilic functionalized PEG/MuCuDCNPs@GOx showed remarkable stability in aqueous solution without aggregation and exhibited zero photo-bleaching. Then the imaging experiments were conducted on mice bearing the subcutaneous xenograft tumor. We systemic administrated PEG/MuCuDCNPs@GOx (5 g L^-1^) through tail vein injection. As Figure 6A shows, the NIR-IIb fluorescence signals of tumor could be detected only after just 3 h post-injection and the signal gradually increased and reached saturation at 12 h post-injection. To further validate its sensitivity, we further attempted to test its imaging effect in tiny tumor. About three days after implantation, a tiny tumor with volume of *c.a.* 1 mm^3^ was successfully established, which was confirmed by the bioluminescence experiment and HE staining (Figure S14). Benefiting from the bright luminescence and the high sensitivity of PEG/MnCuDCNPs@GOx, its fluorescence allowed to detect the early NSCLC tumor with the size only small as *c.a.* 1 mm^3^ (Figure 6B). A SBR of 2.18 was obtained by measuring the line profile across the dot, and the fitting curve also substantiated that the length of tumor was *c.a.* 1 mm (Figure 6C). The *in vivo* imaging results demonstrate the superiority of brightness of NIR-IIb emission to detect 1 mm^3^ early tumor, confirming the increased sensitivity and improved fidelity, as compared to the early-tumor imaging of NIR-I emission 13-14. The efficient diagnosis of lung micro tumors offers chances to in-time inhibit the rapid growth, infiltration and metastasis of early-stage NSCLC 6. Considering the standard first-line treatment for early non-metastatic NSCLC is surgery, we performed the NIR-II guided tumor excision surgery, at 12 h post-injection of PEG/MnCuDCNPs@GOx, the microtumor was removed carefully by visualizing tumor excision under the NIR-IIb fluorescence set-up (1300 LP, 200 ms). The NIR-II imaging exhibited very clear distinction between tumor tissues and adjacent normal healthy tissues (Figure 6D). After completing the *in vivo* imaging, the mice were sacrificed and we extracted the major organs carefully to observe the *in vivo* and* ex vivo* images of the relative biodistribution of the NPs. The* ex vivo* imaging of PEG/MnCuDCNPs@GOx shows that the fluorescence signals in major organs mainly concentrated in the liver and spleen (Figure 6E). We also performed blood vessel imaging *in vivo* through the intact mouse by the tail vein injection to show its superior imaging performance. Benefiting from the increased bright NIR-IIb fluorescence of PEG/MnCuDCNPs@GOx, the arterial blood flow tracking and dynamical visualization of the mouse were realized (Figure 6F). An SBR of 1.58 was calculated by measuring the line profile across the thinner abdominal blood vessel (Figure 6G-H) and an another SBR of 4.13 was obtained by plotting the cross-sectional intensity profiles of the leg vessel (Figure 6I-J). Above results indicate that the PEG/MnCuDCNPs@GOx is a promising nanoplatform to assisting in visualizing early tumors and guiding their removal by surgery.

### *In vivo* Antitumor Study

**Figure 7:**
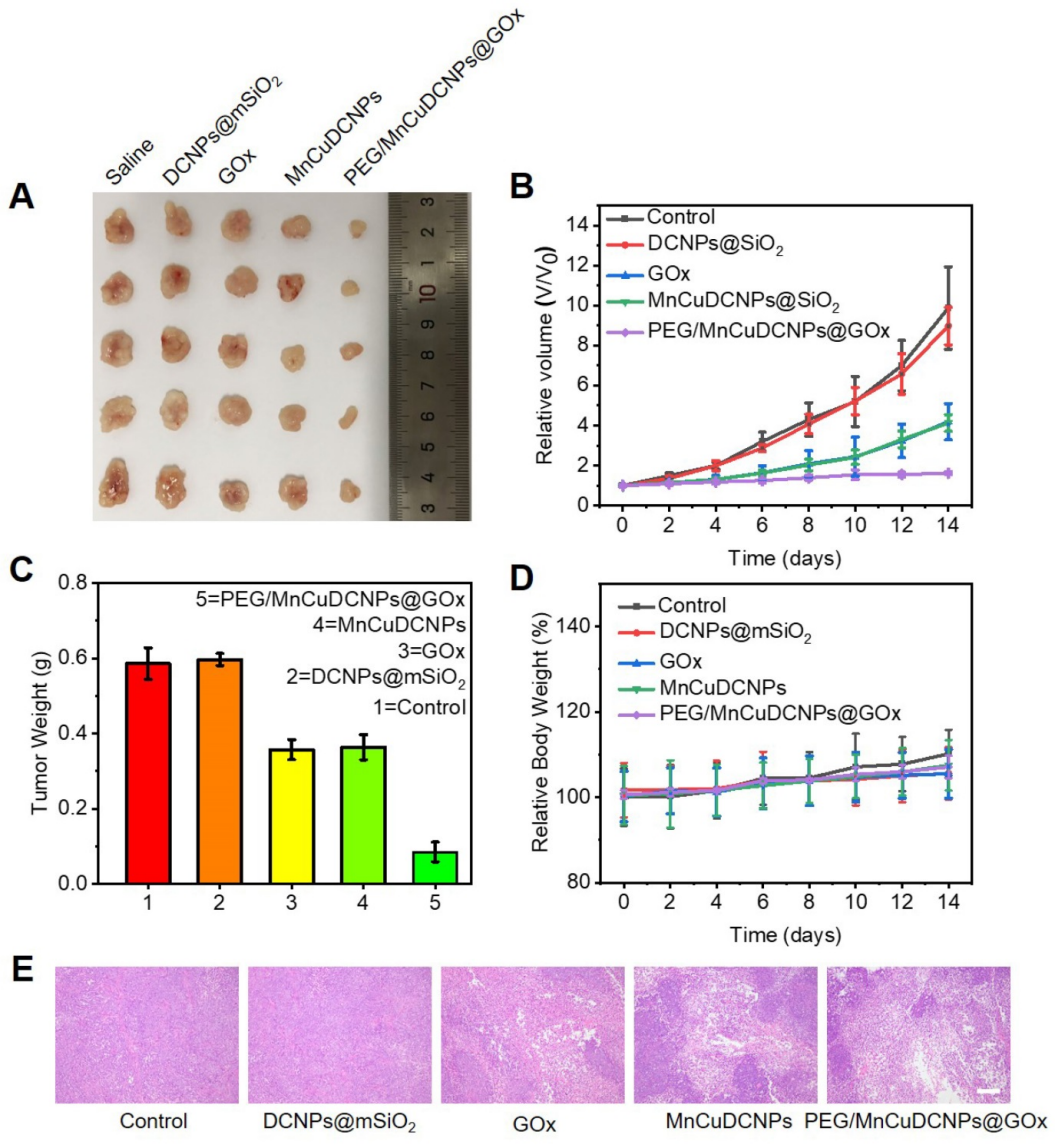
**(A)** Digital photos of tumor tissues excised from the different groups of mice. **(B)** The relative volume of tumor in different groups under 14 days' treatment. **(C)**The corresponding excised tumor weight record of the mice during the 14 days treatment **(D)** Body weights changes of mice in different groups during the 14 days. **(E)** H&E staining of tumor tissues collected in different groups, respectively (scale bars is 100 μm).

Inspired by the anticancer results *in vitro*, the *in vivo* antitumor efficiency of PEG/MnCuDCNPs@GOx was investigated on the A549 tumor subcutaneous xenograft mouse model. We divided the mice with tumor subcutaneous transplantation into five groups to observe the antitumor efficacy of samples after 14 days of treatment. As expected, the PEG/MnCuDCNPs@GOx group achieved the most effective tumor inhibition (Figure 7A). We monitored and recorded changes in tumor volume in each group over the 14-day period. Without any treatment, the tumor volume increased steadily in the control group to 9.8 folds of the original volume, while for the GOx and MnCuDCNPs groups, the number was only 4.2 and 4.1 folds, indicating the efficacy of starvation therapy and CDT *in vivo*, respectively. Moreover, with the treatment of PEG/MnCuDCNPs@GOx, the tumor growth was significantly suppressed, and only a slight increase of 16% in tumor volume was obtained, which demonstrated the synergistic effect of CDT and starvation therapy (Figure 7B). Likewise, the tumor weight was also measured, consistent with result of tumor volume, showing that PEG/MnCuDCNPs@GOx was much more effective in inhibiting tumor growth than other groups were (Figure 7C). In the meantime, almost no body weight was lost during the 14 days (Figure 7D), suggesting the minimum side effects and excellent biocompatibility of these treatments. The hematoxylin and eosin (H&E) stained of tumor tissues of samples treated mice further substantiated the stronger therapeutic efficacy by pointing out the fact that PEG/MnCuDCNPs@GOx actually induced more apoptosis/death than the other groups did in the tumor tissue (Figure 7E). Further, the main organs of the different groups exhibit no histopathological abnormalities, which confirms the biocompatibility of the synthetic sample (Figure S15). Above results clearly substantiate the great promise of PEG/MnCuDCNPs@GOx to serve as an efficient cancer nanotheranostic agent *in vivo*.

## Conclusion

To the best of our knowledge, we firstly demonstrated that NIR-IIb fluorescence diagnosis and image-guided synergistic surgery/starvation/chemodynamic therapeutics is efficient for NSCLC treatment. For the theranostic nanoplatform, PEG/MnCuDCNPs@GOx, by adjusting the doping concentration of the Ce^3+^ in the core and Yb^3+^ in the middle layer of DCNPs (β-NaYF_4_:Yb/Er/Ce@NaYbF_4_@NaYF_4_), ultrabright NIR-IIb emission of 20.3-fold increase was achieved under 980 nm laser excitation. By virtue of the high detection sensitivity, the bright NIR-II fluorescence of PEG/MnCuDCNPs@GOx successfully delineated the xenograft NSCLC tumor with the volume as small as *c.a.* 1 mm^3^ with a SBR of 2.18, and assisted in the intraoperative NIR-II imaging of mice bearing early-stage NSCLC tumors. Furthermore, the DCNPs were decorated with mesoporous silica to load GOx and Mn^2+^Cu^2+^ to fulfill therapeutic functions. The GOx-driven oxidation reaction effectively boosted the Mn^2+^Cu^2+^-mediated Fenton-like reaction for chemodynamic therapy, and cut off the energy supply to starve the tumor cells. Conversely, the MnCuDCNPs could also catalyze H_2_O_2_ decomposition to produce O_2_ to support the starvation therapy. Benefiting from the mutually-supported combinatory therapeutics, the cell survival was suppressed to only 13.98% when cocultured with the PEG/MnCuDCNPs@GOx for 24 h. The tumor growth of the mice transplanted with subcutaneous tumors was more effectively suppressed by PEG/MnCuDCNPs@GOx, compared with groups treated only with GOx or MnCuDCNPs. Simply put, this research demonstrated a novel synergistic theranostic nanoplatform of NSCLC, which might open up a new avenue for the diagnosis and treatment of NSCLC.

## Supplementary Material

Supplementary materials and methods, figures.

## References

[1] Siegel RL, Miller KD, Fuchs HE, Jemal A (2021). Cancer Statistics. CA Cancer J Clin.

[2] Siegel RL, Fedewa SA, Miller KD, Goding-Sauer A, Pinheiro PS, Martinez-Tyson D (2015). Cancer statistics for Hispanics/Latinos. CA Cancer J Clin.

[3] Chen Z, Fillmore CM, Hammerman PS, Kim CF, Wong KK (2014). Non-small-cell lung cancers: a heterogeneous set of diseases. Nat Rev Cancer.

[4] Herbst RS, Morgensztern D, Boshoff C (2018). The biology and management of non-small cell lung cancer. Nature.

[5] Rotow J, Bivona T G (2017). Understanding and targeting resistance mechanisms in NSCLC. Nat Rev Cancer.

[6] Chaft JE, Rimner A, Weder W, Azzoli CG, Kris MG, Cascone T (2021). Evolution of systemic therapy for stages I-III non-metastatic non-small-cell lung cancer. Nat Rev Clin Oncol.

[7] Oudkerk M, Liu SY, Heuvelmans MA, Walter JE, Field JK (2021). Lung cancer LDCT screening and mortality reduction - evidence, pitfalls and future perspectives. Nat Rev Clin Oncol.

[8] Chen R, Manochakian R, James L, Azzouqa AG, Shi H, Zhang Y (2020). Emerging therapeutic agents for advanced non-small cell lung cancer. J Hematol Oncol.

[9] Kim M M, Parolia A, Dunphy MP, Venneti S (2016). Non-invasive metabolic imaging of brain tumours in the era of precision medicine. Nat Rev Clin Oncol.

[10] Kircher MF, Zerda A, Jokerst JV, Zavaleta CL, Kempen PJ, Mittra E (2012). A brain tumor molecular imaging strategy using a new triple-modality MRI-photoacoustic-Raman nanoparticle. Nat Med.

[11] Cao C, Wang X, Cai Y, Sun L, Tian L, Wu H (2014). Targeted *In vivo* Imaging of Microscopic Tumors with Ferritin- based Nanoprobes Across Biological Barriers. Adv Mater.

[12] Sheng Z, Guo B, Hu D, Xu S, Wu W, Liew WH (2018). Bright Aggregation-Induced-Emission Dots for Targeted Synergetic NIR-II Fluorescence and NIR-I Photoacoustic Imaging of Orthotopic Brain Tumors. Adv Mater.

[13] Tao Z, Dang X, Huang X, Muzumdar MD, Xu ES, Bardhan NM (2017). Early tumor detection afforded by *in vivo* imaging of near-infrared II fluorescence. Biomaterials.

[14] Huang S, Lin C-W, Qi J, Iyer A M, He Y, Li Y (2021). Surface Plasmon-Enhanced Short-Wave Infrared Fluorescence for Detecting Sub-Millimeter-Sized Tumors. Adv Mater.

[15] Guo B, Feng Z, Hu D, Xu S, Middha E, Pan Y (2019). Precise Deciphering of Brain Vasculatures and Microscopic Tumors with Dual NIR-II Fluorescence and Photoacoustic Imaging. Adv Mater.

[16] Liu Y, Li Y, Koo S, Sun Y, Liu Y, Liu X (2022). Versatile Types of Inorganic/Organic NIR-IIa/IIb Fluorophores: From Strategic Design toward Molecular Imaging and Theranostics. Chem Rev.

[17] Lu S, Ke J, Li X, Tu D, Chen X (2021). Luminescent nano-bioprobes based on NIR dye/lanthanide nanoparticle composites. Aggregate.

[18] Zhu S, Tian R, Antaris AL, Chen X, Dai H (2019). Near-Infrared-II Molecular Dyes for Cancer Imaging and Surgery. Adv Mater.

[19] Zhang L, Liu Y, Huang H (2022). Multifunctional nanotheranostics for near infrared optical imaging-guided treatment of brain tumors. Adv Drug Deliv Rev.

[20] He Z, Zhang C, Lei Y, Song G, Yao Y (2022). Plasmonic nanomaterials: A versatile phototheranostic platform of cancers. Mater Today.

[21] Zhong Y, Ma Z, Zhu S, Yue J, Zhang M, Antaris A L (2017). Boosting the down-shifting luminescence of rare-earth nanocrystals for biological imaging beyond 1500 nm. Nat Commun.

[22] Zhong Y, Ma Z, Wang F, Wang X, Yang Y, Liu Y (2019). *In vivo* molecular imaging for immunotherapy using ultra-bright near-infrared-IIb rare-earth nanoparticles. Nat Biotechnol.

[23] Zhang H, Fan Y, Pei P, Sun C, Lu L, Zhang F (2019). Tm^3+^-Sensitized NIR-II Fluorescent Nanocrystals for *In vivo* Information Storage and Decoding. Angew Chem Int Ed Engl.

[24] Liu Z, Yun B, Han Y, Jiang Z, Zhu H, Ren F (2022). Dye-Sensitized Rare Earth Nanoparticles with Up/Down Conversion Luminescence for On-Demand Gas Therapy of Glioblastoma Guided by NIR-II Fluorescence Imaging. Adv Healthc Mater.

[25] Ding S, Lu L, Fan Y, Zhang F (2020). Recent progress in NIR-II emitting lanthanide-based nanoparticles and their biological applications. J Rare Earths.

[26] Zhu X, Zhang J, Liu J, Zhang Y (2019). Recent Progress of Rare-Earth Doped Upconversion Nanoparticles: Synthesis, Optimization, and Applications. Adv Sci.

[27] Wang M, Chang M, Li C, Chen Q, Hou Z, Xing B (2022). Tumor-Microenvironment-Activated Reactive Oxygen Species Amplifier for Enzymatic Cascade Cancer Starvation/Chemodynamic /Immunotherapy. Adv Mater.

[28] Xu J, Shi R, Chen G, Dong S, Yang P, Zhang Z (2020). All-in-One Theranostic Nanomedicine with Ultrabright Second Near-Infrared Emission for Tumor-Modulated Bioimaging and Chemodynamic/Photodynamic Therapy. ACS Nano.

[29] Zhang D, Peng R, Liu W, Donovan M, Wang L, Ismail I (2021). Engineering DNA on the Surface of Upconversion Nanoparticles for Bioanalysis and Therapeutics. ACS Nano.

[30] Wang Y, Li Y, Zhang Z, Wang L, Wang D, Tang B (2021). Triple-Jump Photodynamic Theranostics: MnO_2_ Combined Upconversion Nanoplatforms Involving a Type-I Photosensitizer with Aggregation-Induced Emission Characteristics for Potent Cancer Treatment. Adv Mater.

[31] Wang Z, Liu B, Sun Q, Feng L, He F, Yang P (2021). Upconverted Metal-Organic Framework Janus Architecture for Near-Infrared and Ultrasound Co-Enhanced High Performance Tumor Therapy. ACS Nano.

[32] Guo B, Wu M, Shi Q, Dai T, Xu S, Jiang J (2020). All-in-One Molecular Aggregation-Induced Emission Theranostics: Fluorescence Image Guided and Mitochondria Targeted Chemo-and Photodynamic Cancer Cell Ablation. Chem Mater.

[33] Wang S, Tian R, Zhang X, Cheng G, Yu P, Chang J (2021). Beyond Photo: Xdynamic Therapies in Fighting Cancer. Adv Mater.

[34] Zhou Y, Fan S, Feng L, Huang X, Chen X (2021). Manipulating Intratumoral Fenton Chemistry for Enhanced Chemodynamic and Chemodynamic-Synergized Multimodal Therapy. Adv Mater.

[35] Liang C, Zhang X, Yang M, Dong X (2019). Recent Progress in Ferroptosis Inducers for Cancer Therapy. Adv Mater.

[36] Li C, Ye J, Yang X (2022). Fe/Mn Bimetal-Doped ZIF-8-Coated Luminescent Nanoparticles with Up/Downconversion Dual-Mode Emission for Tumor Self-Enhanced NIR-II Imaging and Catalytic Therapy. ACS Nano.

[37] Xu J, Wang J, Ye J, Jiao J, Liu Z, Zhao C, Li B, Fu Y (2021). Metal-Coordinated Supramolecular Self-Assemblies for Cancer Theranostics. Adv Sci.

[38] Yang B, Chen Y, Shi J (2019). Nanocatalytic Medicine. Adv Mater.

[39] Fu LH, Qi C, Hu YR, Lin J, Huang P (2020). Glucose Oxidase-Instructed Multimodal Synergistic Cancer Therapy. Adv Mater.

[40] Fu LH, Wan Y, Qi C, He J, Li C, Yang C (2021). Nanocatalytic Theranostics with Glutathione Depletion and Enhanced Reactive Oxygen Species Generation for Efficient Cancer Therapy. Adv Mater.

[41] Ma B, Wang S, Liu F, Zhang S, Duan J, Li Z (2019). Self-Assembled Copper-Amino Acid Nanoparticles for *in situ* Glutathione "AND" H2O2 Sequentially Triggered Chemodynamic Therapy. J Am Chem Soc.

[42] Jana D, Zhao Y (2022). Strategies for enhancing cancer chemodynamic therapy performance. Exploration.

[43] Xiong Y, Xiao C, Li Z, Yang X (2021). Engineering nanomedicine for glutathione depletion-augmented cancer therapy. Chem Soc Rev.

[44] Li C, Wan Y, Zhang Y, Fu L, Blum N, Cui R (2022). *In situ* Sprayed Starvation/Chemodynamic Therapeutic Gel for Post-Surgical Treatment of IDH1 (R132H) Glioma. Adv Mater.

[45] Feng L, Xie R, Wang C, Gai S, He F, Yang D (2018). Magnetic Targeting, Tumor Microenvironment-Responsive Intelligent Nanocatalysts for Enhanced Tumor Ablation. ACS Nano.

[46] Lin L S, Song J, Song L, Ke K, Liu Y, Zhou Z (2018). Simultaneous Fenton-like Ion Delivery and Glutathione Depletion by MnO_2_ -Based Nanoagent to Enhance Chemodynamic Therapy. Angew Chem Int Ed Engl.

[47] Wang Y, Zhou S, Sun F, Hu P, Zhong W, Fu J (2022). In-depth insight into the Yb^3+^ effect in NaErF_4_-based host sensitization upconversion: a double-edged sword. Nanoscale.

[48] Kuang Y, Li TY, Jia T, Gulzar A, Zhong C, Gai S (2020). Insight into the Luminescence Alternation of Sub-30 nm Upconversion Nanoparticles with a Small NaHoF_(4)_Core and Multi-Gd_3+_/Yb_3+_ Coexisting Shells. Small.

[49] Wurth C, Fischer S, Grauel B, Alivisatos A, Resch-Genger U (2018). Quantum Yields, Surface Quenching, and Passivation Efficiency for Ultrasmall Core/Shell Upconverting Nanoparticles. J Am Chem Soc.

[50] Zhang K, Xu LL, Jiang JG, Calin N, Lam KF, Zhang SJ (2013). Facile Large-Scale Synthesis of Monodisperse Mesoporous Silica Nanospheres with Tunable Pore Structure. J Am Chem Soc.

[51] Yu Q, Han Y, Wang X, Qin C, Zhai D, Yi Z (2018). Copper Silicate Hollow Microspheres-Incorporated Scaffolds for Chemo-Photothermal Therapy of Melanoma and Tissue Healing. ACS Nano.

[52] Wang S, Li F, Qiao R, Hu X, Liao H, Chen L (2018). Arginine-Rich Manganese Silicate Nanobubbles as a Ferroptosis-Inducing Agent for Tumor-Targeted Theranostics. ACS Nano.

[53] Sun X, Du F (2015). Synthesis under mild conditions and high catalytic property of bimetal Ni-Cu/SiO2 hollow spheres. RSC Adv.

[54] Hao H, Sun M, Li P, Sun J, Liu X, Gao W (2019). *In situ* Growth of a Cationic Polymer from the N-Terminus of Glucose Oxidase To Regulate H_2_O_2_ Generation for Cancer Starvation and H_2_O_2_ Therapy. ACS Appl Mater Interfaces.

[55] Borresen B, Henriksen J, Clergeaud G, Jorgensen J, Melander F, Elema D (2018). Theranostic Imaging May Vaccinate against the Therapeutic Benefit of Long Circulating PEGylated Liposomes and Change Cargo Pharmacokinetics. ACS Nano.

[56] Liu C, Wang D, Zhang S, Cheng Y, Yang F, Xing Y (2019). Biodegradable Biomimic Copper/Manganese Silicate Nanospheres for Chemodynamic/Photodynamic Synergistic Therapy with Simultaneous Glutathione Depletion and Hypoxia Relief. ACS Nano.

[57] Dong H, Liu C, Ye H, Hu L, Fugetsu B, Dai W (2015). Three-dimensional Nitrogen-Doped Graphene Supported Molybdenum Disulfide Nanoparticles as an Advanced Catalyst for Hydrogen Evolution Reaction. Sci Rep.

[58] Tao Y S, Kanoh H, Abrams L, Kaneko K (2006). Mesopore-modified zeolites: Preparation, characterization, and applications. Chem Rev.

