# Supplementary material for: NIR-IIb fluorescence-image guided synergistic surgery/starvation/chemodynamic therapy: an innovative treatment paradigm for malignant non-small cell lung cancers
Source: Theranostics. 2023 Apr 1;13(7):2176–91. doi: 10.7150/thno.83753 (PMC10157733; doi:10.7150/thno.83753)
Supplement: Supplementary file 1 — Supplementary materials and methods, figures. [file thnov13p2176s1.pdf]

Supplementary data

NIR-IIb fluorescence-image guided synergistic surgery/starvation/chemodynamic therapy: an innovative treatment paradigm for malignant non-small cell lung cancers

Xuejiao Han[1,2#], Yingtao Zhong[2#], Chao Mi[3], Zhiguo He[2], Jingsi Gu[4], Xiaoyong Dai[5], Chenguang Ma[3], Chunyan Feng[5], Huaqing Chen[5], Zebin Lan[2], Zhiyong Guo[3], Laiqiang Huang[5], Baozhu Zhang[6], Bing Guo[2*], Qingwei Meng[1*]

Experimental Section/Methods

1. Materials

$Y(CH_3COO)_3 \cdot 6H_2O$ (99.9%), $Yb(CH_3COO)_3 \cdot 6H_2O$ (99.9%), $Ce(CH_3COO)_3 \cdot 6H_2O$ (99.9%), $Er(CH_3COO)_3 \cdot 6H_2O$ (99.5%), oleic acid (OA, 85%), ammonium fluoride (NH_4F, 98%), tetraethyl orthosilicate (TEOS, 98%), glutathione were acquired from Aladdin Chemical Technology Co., Ltd., (Shanghai, China). 1-octadecene (ODE, >90%), sodium hydroxide (NaOH, 96%), cetyltrime-thylammonium bromide (CTAB), ammonium nitrate (NH_4NO_3) and ammonium hydroxide ($NH_3 \cdot H_2O$, 28%) were acquired from Macklin Chemical Reagents. All materials were used as received.

2. Characterization

Transmission electron microscopy (TEM) images were obtained by a JEM-1400 microscope (JEOL, Japan). The scanning transmission electron microscopy (STEM) micrograph and energy-dispersive spectroscopy (EDS) were conducted by an S-4800 microscope (Hitachi, Japan). Fluorescence spectra of downconversion nanoparticles were recorded by a spectrofluorometer (Fluoro Max-4). DLS test and ζ-potential were conducted on Malvern Nanoziser (Zetasizer Nano ZSP) at ambient temperature. Powder X-ray diffraction (XRD) analysis was conducted on a D8 Focus diffractometer (SmartLab3KW,

Japan). The UV-vis absorption spectra were recorded by Uv-vis spectrophotometer (UV-2600, SHIMADZU). Fourier transform infrared (FT-IR) spectra were recorded using a Vertex 70 spectrophotometer (Bruker, Germany). Confocal laser scanning microscopy (CLSM) images were obtained using a STELLARIS 8 confocal microscope (Leica, Germany).

3. Synthesis of β-$NaYF_4$:Yb/Er/Ce@$NaYF_4$:Yb@$NaYF_4$

1 mmol rare earth acetylized (RE: 30% Yb, 10% Ce, 2% Er, 58%Y) were added to a three-necked flask containing of 6.5 mL oleic acid (OA) and 15 mL 1-octadecene (ODE) and heated to 100 °C with stirring vigorously. Continuously heating to 125 °C under vacuum and keep 20-30 minutes until the rare earth acetylized is completely dissolved, a lightly yellow transparent solution was obtained. After the solution naturally cooled down to 48°C, 2.5 mmol NaOH (about 100 mg) and 4mmol NH_4F (about 148.5 mg) were dissolved in methanol respectively, then quickly mixed and added into the three-necked flask, and vigorously stirred for 1 h until the solution turned from turbid white to clarified yellow. The mixture was then protected by the argon gas atmosphere and heated to 305 °C for 1h. The precipitated product was added with an equal amount of anhydrous ethanol and centrifuged at 5000rpm for 10min, then re-dispersed in 5 mL of cyclohexane.

$Yb(CH_3COO)_3 \cdot 6H_2O$ (1 mmol) was dispersed in a mixture of 6.5 mL OA and 15 mL ODE, similar to the above, the solution was heated to 100 °C with stirring vigorously and then continuously raised the temperature to 125 °C under vacuum to completely dissolve. Differently, after cooled down to 100 °C, the core (β-$NaYF_4$:Yb/Er/Ce) were dropped into the solution. The following process was similar to the process of core (β-$NaYF_4$: Er/Ce/Yb) mentioned above.

We used the same β-$NaYF_4$:Er/Ce/Yb@$NaYbF_4$ core-shell nanoparticle to synthesize β-$NaYF_4$:Yb/Er/Ce@$NaYbF_4$@$NaYF_4$ core-shell-shell nanoparticles with different shell thicknesses. Firstly, the $Y(CH_3COO)_3·6H_2O$ (2.5 mmol), OA (10.0 mL), and ODE (15.0 mL) were put into a flask and heated at 140 °C under pumping with stirring for 1 hour to get rid of residual water and oxygen. Then the 0.10 M Y-OA precursor solution was received. The same core-shell nanoparticle batch was used to minimize any differences due to effects of the size, morphology, lanthanide doping, or any other variation of the core-shell that may occur between multiple synthesis. We followed the synthesis and sequential shelling procedure as described in literature.1. Briefly, 0.5 mmol of β-$NaYF_4$:Er/Ce/Yb@$NaYbF_4$ core NPs were added three-neck flask containing 4.0 mL of OA and 6.0 mL of ODE. The solution was pumped down at 80 °C for 30 min to remove cyclohexane. Then, the system was heated to 300 °C under the argon gas atmosphere with vigorous stirring for 20 min. Subsequently, the 1.0 ml of Y-OA precursors were injected the above solution maintained for 15 min respectively. The shell thickness was tuned by the injection times. Finally, the resulting NPs were precipitated by addition of ethanol, and redispersed in cyclohexane. The obtained core−shell−shell (β-$NaYF_4$:Yb/Er/Ce@$NaYbF_4$ @$NaYF_4$) DCNPs were marked as DCNPs.

4. Synthesis of Mesoporous Silica-Coated DCNPs (DCNPs@mSiO_2)

CTAB (0.1 g) was dispersed in 20 mL deionized water by continuously stirred treating until the solution is clear. Then added 2 mL of the DCNPs into the solution. The CTAB-DCNPs solution was placed in a round-bottom flask, and 40 mL deionized water, 300 μL NaOH (2 M), and 6 mL ethanol were added, respectively. The temperature was slowly raised to 70 °C with stirring. After the temperature stabilized, add 250 μL TEOS slowly to the solution, stirring for 1 h, centrifuging (8000 RPM, 10 min) to obtain white product DCNPs@mSiO_2. Anhydrous ethanol was used to wash three times (8000 RPM, 10 min) to remove the unreacted TEOS. The cleaned DCNPs@mSiO_2 was re-dispersed in anhydrous ethanol, then

0.3g ammonium nitrate (NH_4NO_3) was added and raise the temperature to 70 °C. The reflux stirring reaction was performed for 2 h. The final product was centrifuged with anhydrous ethanol for three times (8000 RPM, 10 min) to obtain DCNPs@mSiO_2.

5. Synthesis of Manganese/Copper Silicate Coated DCNPs (MnCu@DCNPs)

First, 50 mg of DCNPs@mSiO_2 was dispersed in 20 mL of aqueous solution, and 0.4 mmol of $MnCl_2 \cdot 4H_2O$ was added. After that, NH_4Cl (10 mmol) and $Cu(NO_3)_2 \cdot 3H_2O$ (0.4 mmol) were dissolved in another 20 mL of aqueous solution, and 1 mL of $NH_3 \cdot H_2O$ (28%) was further added. These two solutions were then mixed and transferred to the Teflon-lined autoclave, reacted at 180°C for 1h, and centrifuged to collect the product.

6. Synthesis of MnCuDCNPs@GOx

Briefly, 5 mg mL^{-1} of GOx were dissolved in the obtained MnCu@DCNPs and stirred overnight. Then the MnCuDCNPs@GOx sample was separated by centrifugation (14000 rpm, 10 min), washed and dried.

7. Synthesis of PEGylated MnCuDCNPs @GOx (PEG/ MnCuDCNPs@GOx)

MnCuDCNPs@GOx were dissolved in 50 mL of deionized water with PAH dispersed in. Afterward, PAHylated MnCuDCNPs@GOx were obtained by centrifugation and washed by deionized water. To obtain PEG/MnCuDCNPs@GOx, PEG-COOH and PAHylated MnCuDCNPs@GOx were dissolved in deionized water, then stirred overnight and centrifuged.

8. Hydroxyl radicals (•OH) generation

In order to investigate the generation of •OH by degraded MnCu@DCNPs, 20 μg mL^{-1} of MnCu@DCNPs was added into a buffer solution containing 25 mM GSH, 10 μg mL^{-1} MB,

and 10 mM H_2O_2, then reacted at 37 °C for 0, 10, 20, 30 min. The solution absorbance was measured at 650 nm.

9. *In Vitro* Degradation Experiments

The PEG/MnCu@DCNPs@GOx suspension (1 mL, 5 mg mL^{-1}) was placed in dialysis bags (MWCO = 3600), and then the dialysis bag was sealed to dialyze against another 39 mL of PBS at pH 7.5, 6.5, and 5.5, respectively, to simulate different biological environments. The degradation experiment was carried out at 37 °C under constant shaking. At each time point, 1 mL of media was taken from the solution outside the bags to measure the concentrations of Cu^{2+} and Mn^{2+} ions, and 6 mL of fresh PBS with the same pH value was added to the solution.

10. Cellular uptake

A549 cells were inoculated for 24 h to obtain a monolayer of cells. Then, FITC-modified PEG/MnCuDCNPs@GOx (1 mL, 0.5 mg/mL) was transferred and cultured for 1 h and 2h, respectively. Afterward, the cells were rinsed with PBS, and DAPI was added to each well to stain the cell nucleus for 15 min. Furthermore, A549 cells were fixed with glutaraldehyde. Finally, the cells were further poached several times with PBS to observe the fluorescence images.

11. Detection of ROS *in vitro*

For ROS observations, A549 cells were treated with PEG/MnCuDCNPs@GOx in high-glucose medium for 2 h. After that, the A549 cells were rinsed and stained with 1.7 μL of DCFH-DA at 37 °C for 30 min. Then, the cells were washed with serum-free DMEM and observed by a Nikon Eclipse Ts2 inverted fluorescence microscope (Nikon Corporation, Japan) under 470 nm wavelength excitation. For live and dead cell observations, the A549

cells were cocultured with at the equivalent concentration of PEG/MnCuDCNPs@GOx in 1000 μg mL^{-1} glucose-containing DMEM media for 24 h. After that, the cells of each group were incubated with CA (4 μM) and PI (4 μM) staining reagents for 10 min and then observed using a Nikon Eclipse Ts2 inverted fluorescence microscope.

12. Cytotoxicity Assay

For *in vitro* cytotoxicity assays, A549 cells were seeded into 96-well plates at a density of 1 × 10^4 cells per well, respectively. After 24 h of incubation, the media of the 96-well plates were discarded. Subsequently, PEG/MnCuDCNPs@GOx and DCNPs@mSiO_2 with different concentrations were dispersed into fresh DMEM containing different concentrations of glucose (0−1000 μg mL^{-1}) and then inoculated into the 96-well plates and cocultured for 12 h. Cell viability was quantified by the cck8 assay and calculated based on the following formula: cell viability = (OD_{450nm} of the sample/OD_{450nm} of the control) × 100%. The cell viability of the control group was set as 100%.

13. Flow cytometry

Flow cytometry was conducted for cell apoptosis analysis. A549 cells were seeded (1×10^5 cells in 1 mL per well) in 6-well plates and cultured for 24 hours. Then the culture media were replaced by saline, DCNPs@mSiO_2, GOx, MnCuDCNPs, and PEG/MnCuDCNPs@GOx in DMEM and incubated for another 24 h. After co-incubation, the cells were collected by centrifugation. Then, the Annexin V-FITC/PI Apoptosis Detection Kit was used to stain the cells for 20 min before the flow cytometry analysis.

14. *In Vivo* NIR-II Imaging with PEG/MnCuDCNPs@GOx

PEG/MnCuDCNPs@GOx particles (DCNPs concentration = 0.5 mg/kg) was I.V. injected into BALB/c nude mice bearing A549 lung cancer subcutaneous tumors. NIR-II images were acquired at different time points using an in vivo imaging system NIR-OPTICS series III

900/1700 small animal imaging system. At 24 h postinjection, mice were euthanized, and the main organs and tumors were collected for ex vivo fluorescence imaging.

15. Antitumor Study

Animals Female BALB nude mice aged 4-5 weeks were purchased from Guangdong Medicinal Laboratory Animal Center (Guangzhou, China) and a xenograft BALB/c nude mice model was built via injecting 5×10^6 of A549 cells into the right axilla of nude mice. The mice were randomly divided into 5 groups (n = 5/group), including (1) saline, (2) DCNPs@mSiO_2, (3) GOx, (4) MnCuDCNPs, and (5) PEG/MnCuDCNPs@GOx. The injection dose for PEG/MnCuDCNPs@GOx in this assay is 100 μL (200 μg/mL). All of the above nanocomposites were intravenously injected every 3 days. The tumor volume and body weight were detected every other day. The maximum length and the minimal width of tumors were measured by digital vernier calipers. The tumor volumes were determined by the following formula: Volume = (tumor length) × (tumor width)^2 /2. The mice were sacrificed and the tumor and major organs were separated for histological analysis after the treatment for 14 days. All the mouse experiments conform to the standards of The National Regulation of China for Care and Use of Laboratory Animals. The animal study was reviewed and approved by the Administrative Committee on Animal Research of the Graduate School at Shenzhen, Tsinghua University.

16. Histological Examination

After 14 days of treatment, the heart, liver, spleen, lung, kidney, and tumors were excised and dehydrated with buffered formalin, ethanol, and xylene in turn. Finally, all types of dehydrated tissues were embedded in liquid paraffin to obtain stained slices for H&E staining by optical microscope.

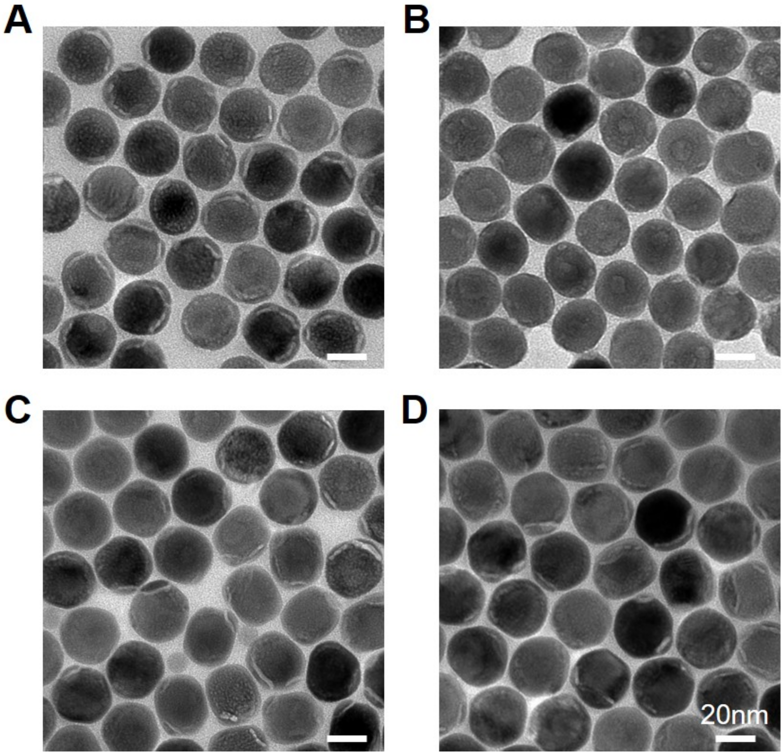

Figure S1. TEM images of as-prepared β-$NaYF_4$:Yb/Er/Ce@$NaYbF_4$@$NaYF_4$ with different $NaYF_4$ shell thicknesses: **(A)** 3.5nm, **(B)** 4.7nm, **(C)** 6.2nm, **(D)** 7.3nm.

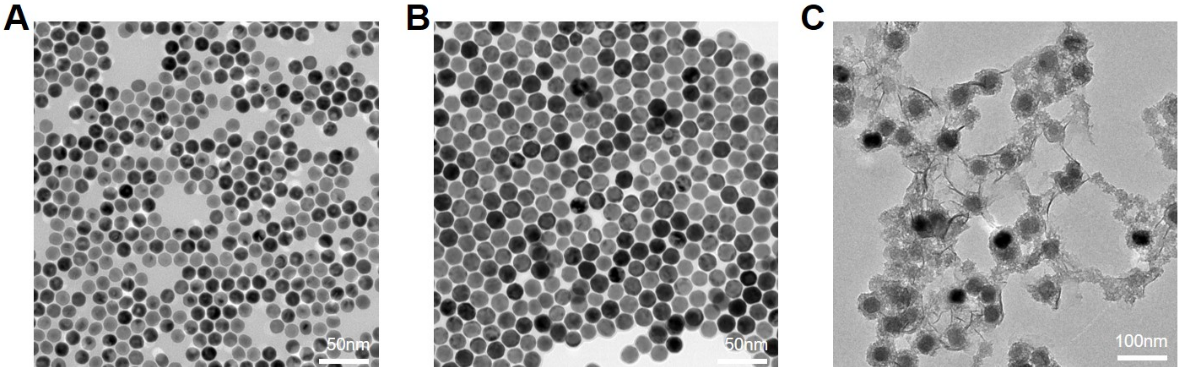

Figure S2. TEM images of **(A)** core (β-$NaYF_4$:Yb/Er/Ce) **(B)** core-shell (β-$NaYF_4$:Yb/Er/Ce@$NaYbF_4$) of DCNPs and **(C)** MnCuDCNPs.

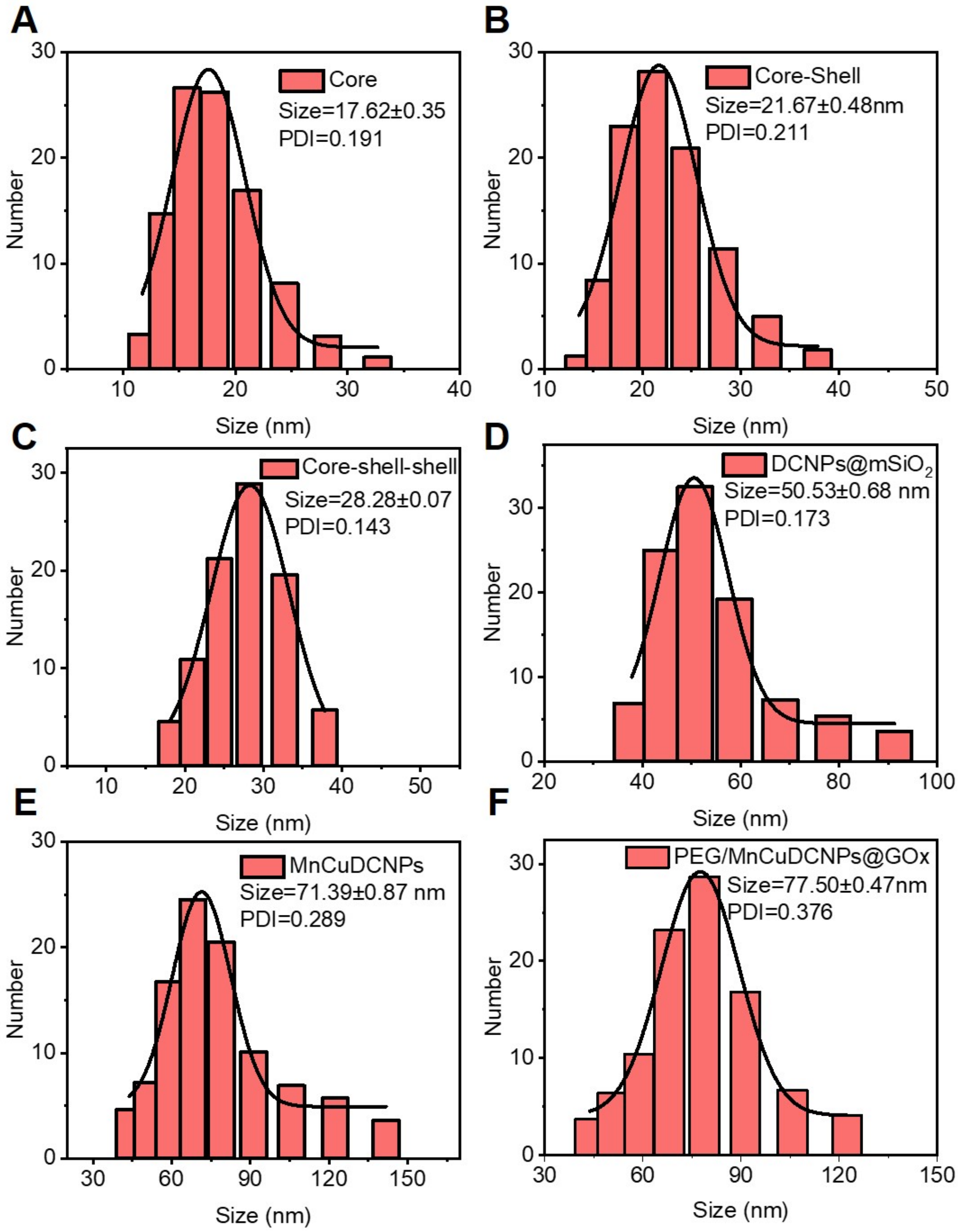

Figure S3. The statistics of particle size of **(A)** β-$NaYF_4$:Yb/Er/Ce, **(B)** β-$NaYF_4$:Yb/Er/Ce@$NaYbF_4$, **(C)** β-$NaYF_4$:Yb/Er/Ce@$NaYbF_4$@$NaYF_4$, **(D)** DCNPs@$mSiO_2$ and **(E)** MnCuDCNPs, **(F)** PEG/MnCuDCNPs@GOx.

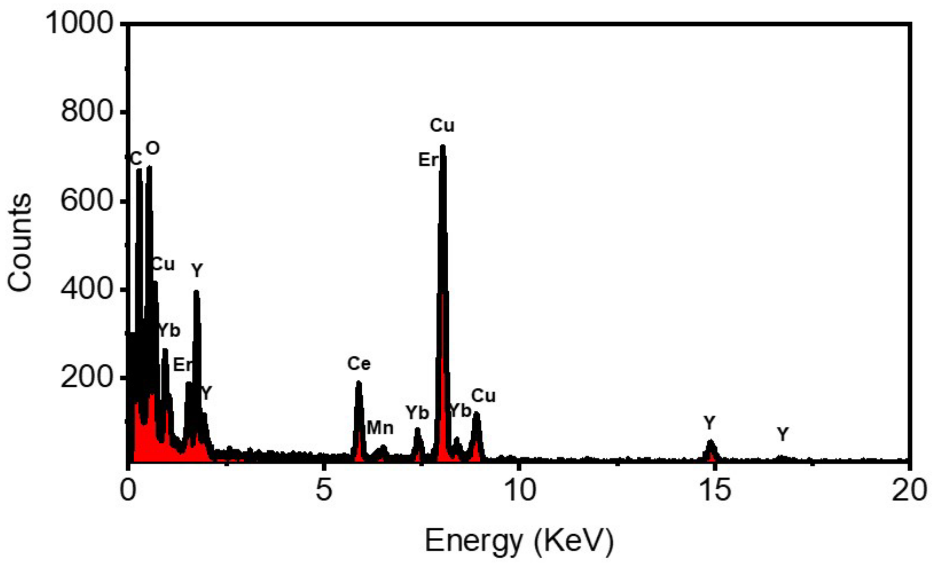

Figure S4. Energy-dispersive spectroscopy (EDS) of PEG/MnCuDCNPs@GOx.

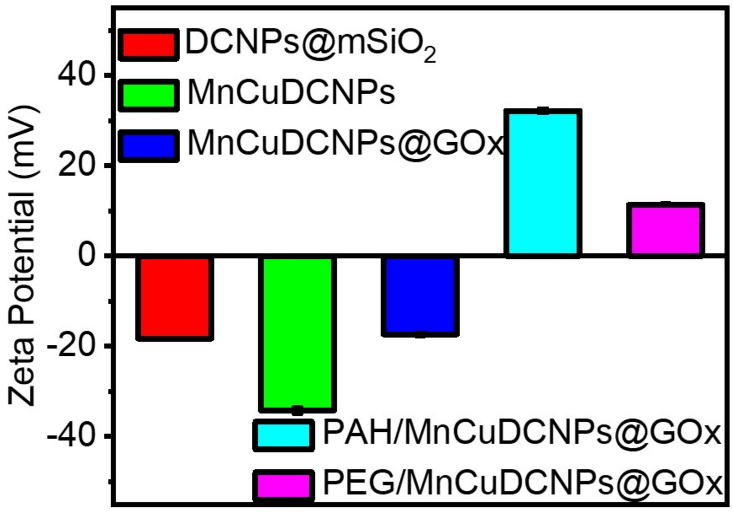

Figure S5. The zeta potential of the samples.

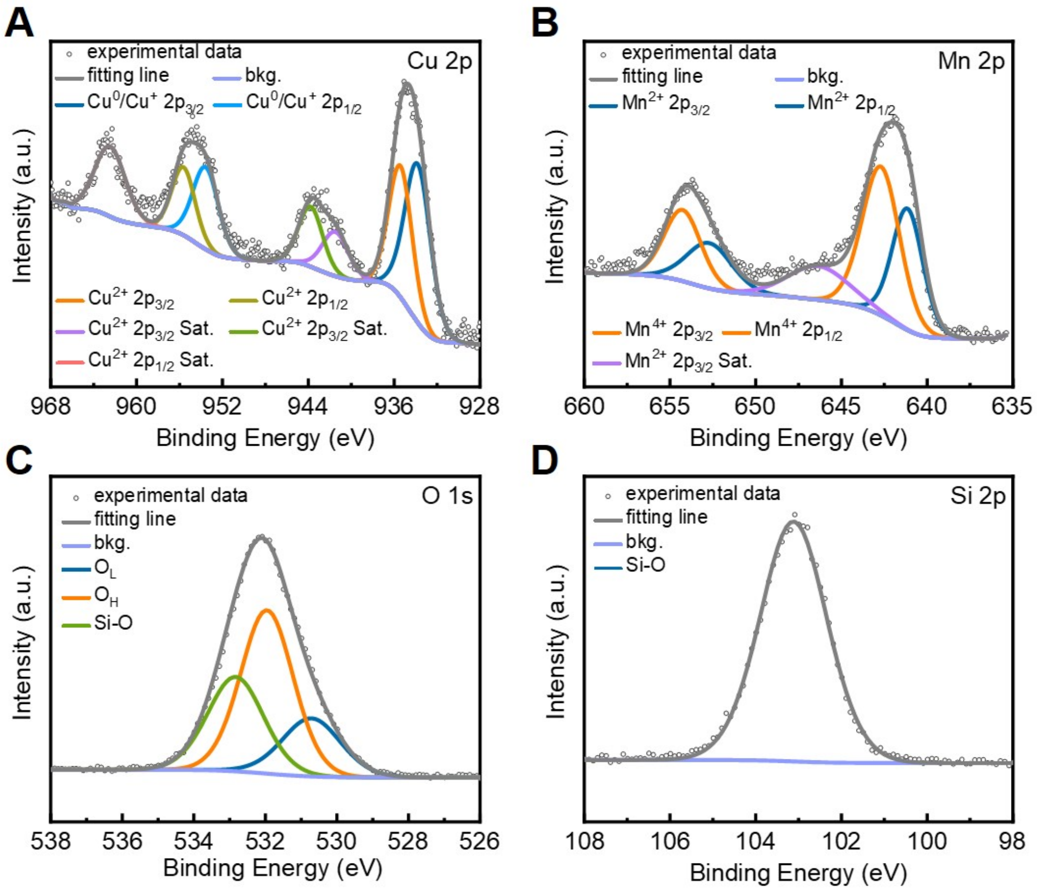

Figure S6. XPS spectra of MnCuDCNPs@GOx: **(A)** High resolution Cu 2p XPS spectrum, which indicates the existence of a Cu^{2+} phase on the MnCuDCNPs@GOx surface. **(B)** High-resolution Mn 2p spectrum of MnCuDCNPs@GOx, fitted to energy components centered at around 654 and 642 eV. **(C)** High-resolution O 1s spectrum of MnCuDCNPs@GOx, fitted to energy component centered at around 531 eV. **(D)** High resolution Si 2p spectrum of MnCuDCNPs@GOx, fitted to energy component centered at around 103 eV.

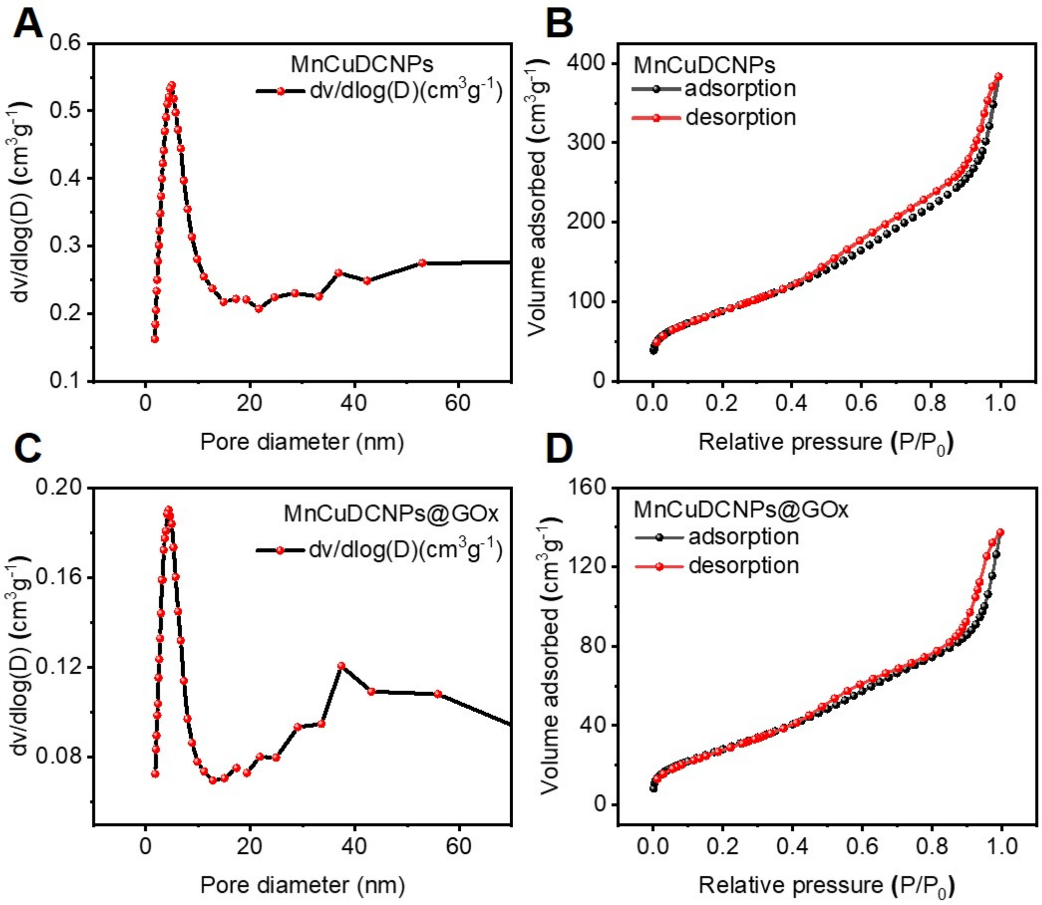

Figure S7. **(A)** N_2 adsorption/desorption curves, **(B)** pore volume distributions of MnCuDCNPs. **(C)** N_2 adsorption/desorption curves, **(D)** pore volume distributions of MnCuDCNPs@GOx.

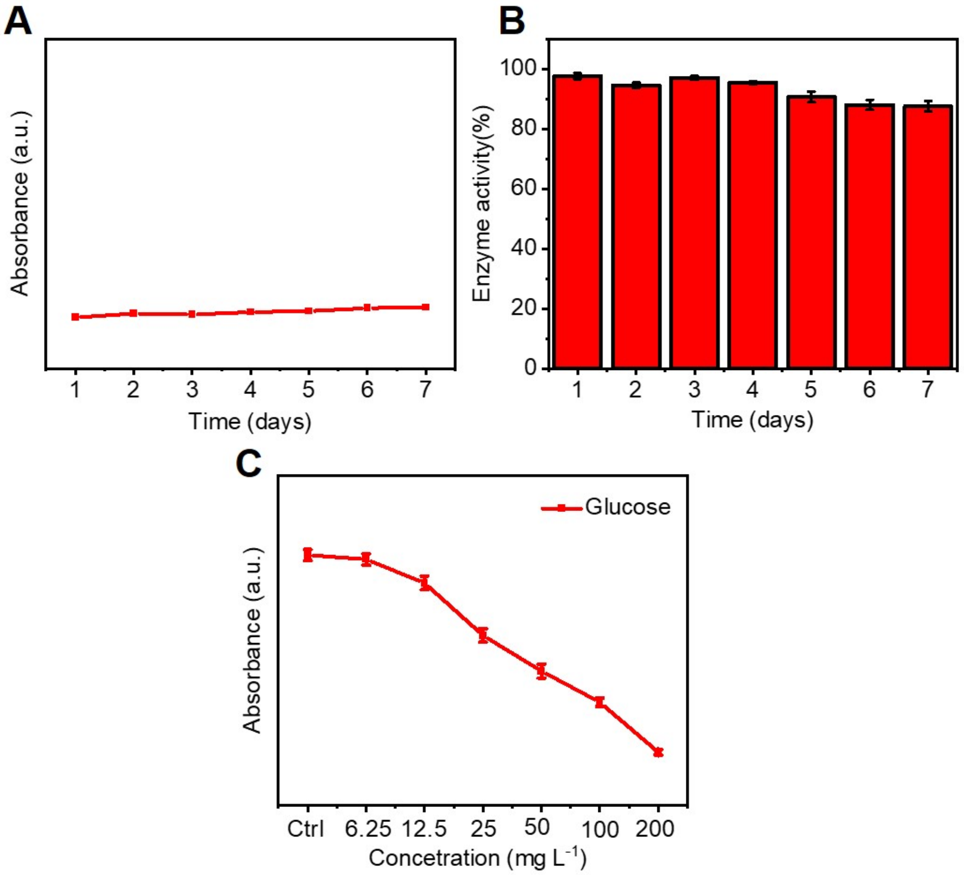

Figure S8. (A) Absorbance value of the BCA test kit corresponding to the detection of GOx concentration in pH=7.4 aqueous solution after the PEG/MnCuDCNPs@GOx stored into for seven days.**(B)** Enzyme activity decay of PEG/MnCuDCNPs@GOx over time at room temperature. **(C)**The absorbance of glucose detection kit for the detection of glucose in vitro under different concentration treatment of PEG/MnCuDCNPs@GOx.

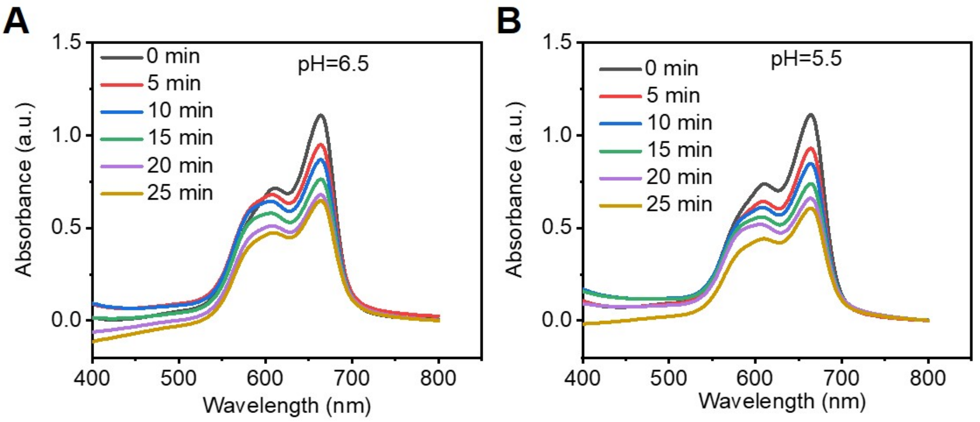

Figure S9. MB degradation curves under •OH radicals which were generated by MnCuDCNPs treated with GSH and H_2O_2 at **(A)** pH = 6.5 and **(B)** pH = 5.5.

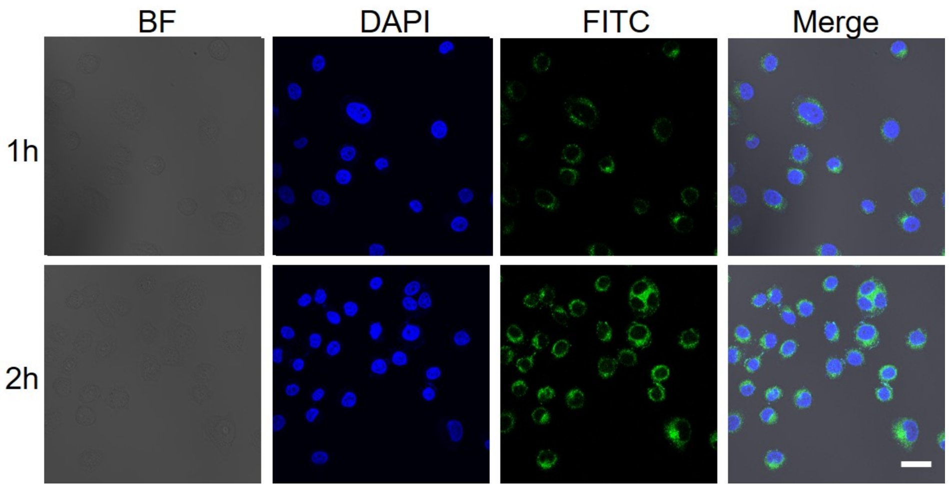

Figure S10. CLSM images of A549 cells after co-incubation with FITC decorated PEG/MnCuDCNPs@GOx for different times. Scale bar: 50 μm.

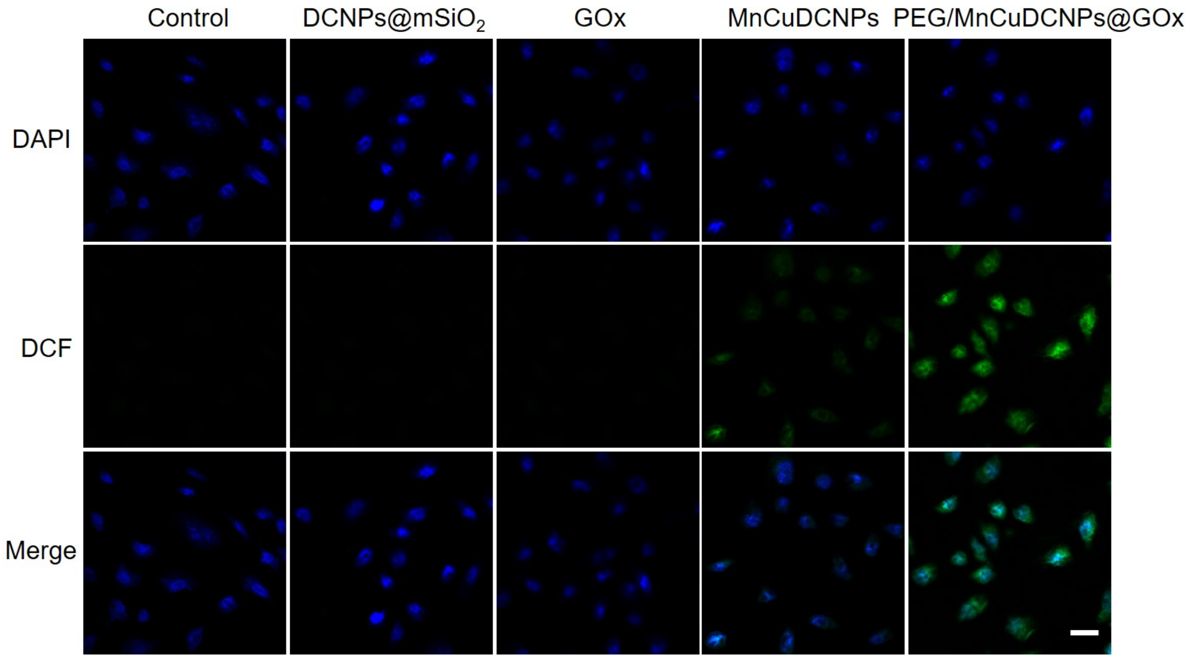

Figure S11. Fluorescent images of the A549 cells incubated with DCNPs@mSiO_2, GOx, MnCuDCNPs and PEG/MnCuDCNPs@GOx. The nuclei were stained with DAPI, and the green fluorescence corresponded to the signals generated by ROS probe of DCF. Scale bar: 50 μm.

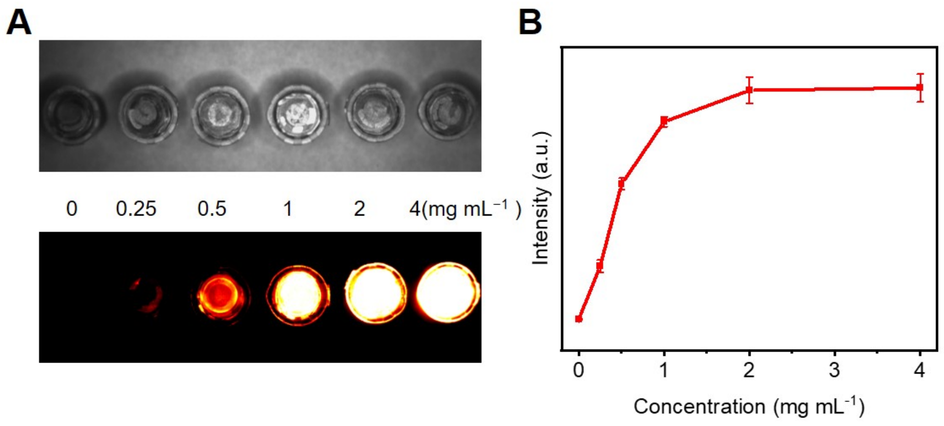

Figure S12. The NIR-II fluorescent images of PEG/MnCuDCNPs@GOx solutions with different concentrations.

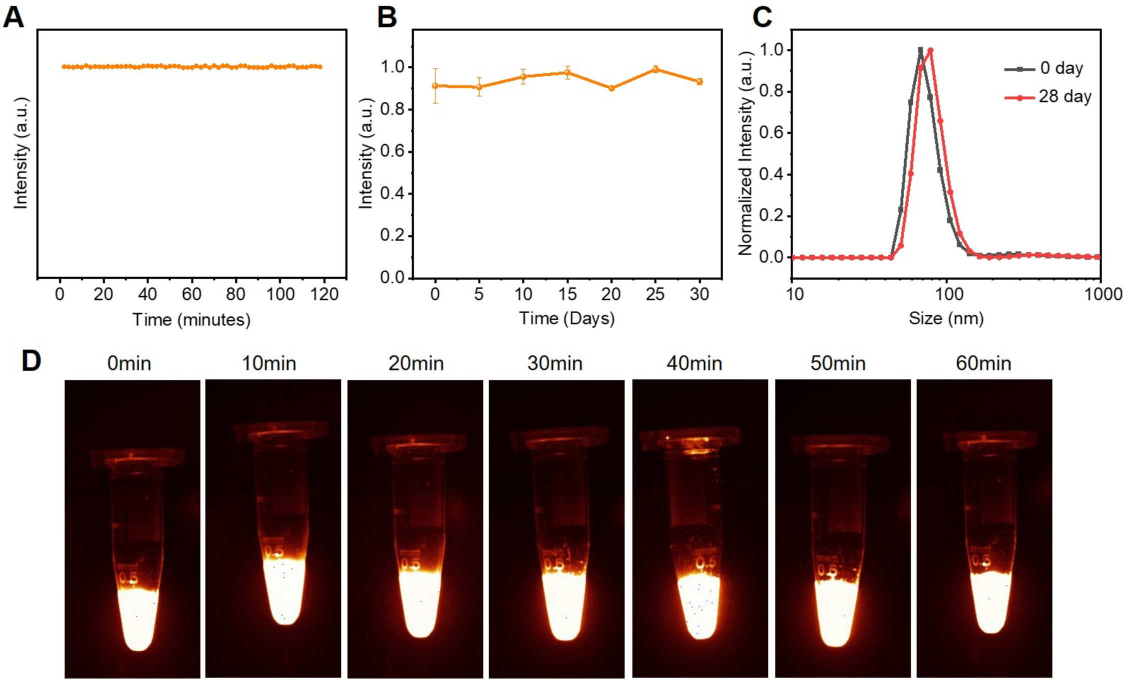

Figure S13. (A) The 1525 nm luminescence intensity of PEG/MnCuDCNPs@GOx as a function of irradiation time (2 hours) excited by a 980 nm laser (power density: 200 mW/cm^2). **(B)** Long-term photo-stability of PEG/MnCuDCNPs@GOx, which was investigated by monitoring the fluorescence intensity changes at different time points. Means ± SD were from three independent experiments. **(C)** DLS data at 0 and 28 day for PEG/MnCuDCNPs@GOx during the storage conditions. **(D)**The imaged of PEG/MnCuDCNPs@GOx in water continuously irradiated with a 980nm laser (38mW cm^{-2}).

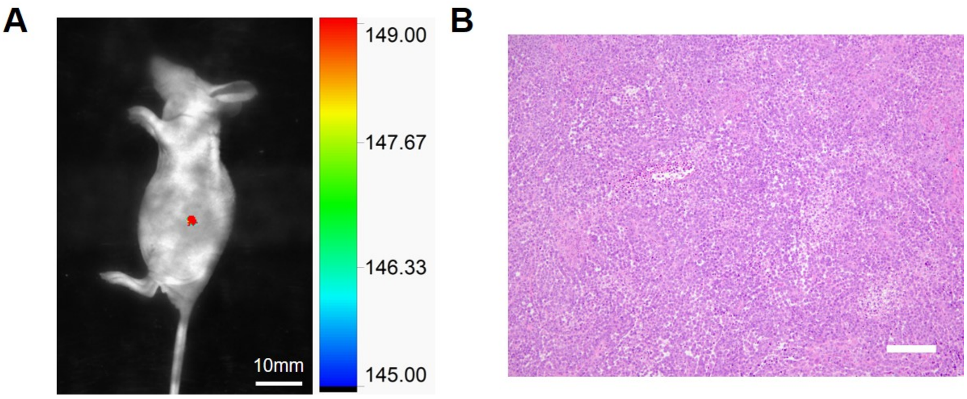

Figure S14. (**A**) *In vivo* bioluminescence image of tumor with the volume of 1 mm^3. **(B)** H&E stained histological sections of the tumor with the volume of 1 mm^3. Scale bar: 100 μm.

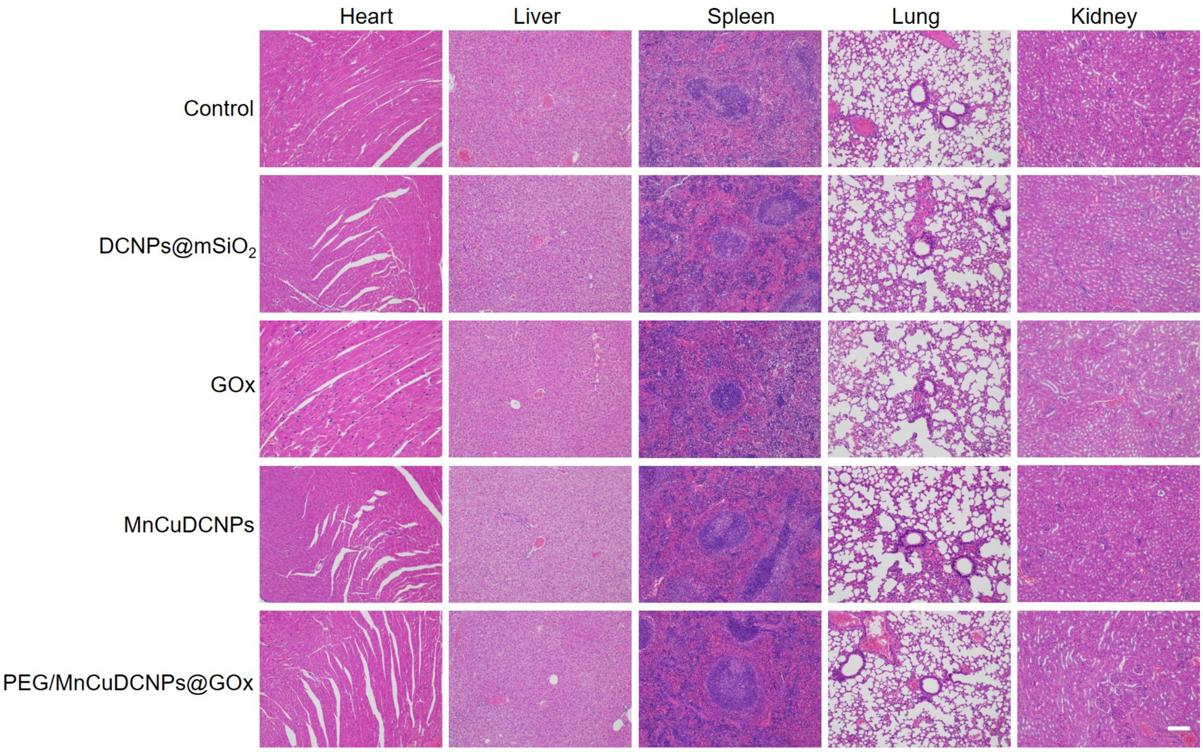

Figure S15. H&E stained histological sections of the major organs (*e.g.*, heart, liver, spleen, lung, and kidney). Scale bar: 100 μm.

Reference

[1] Li M, Shen D, Yang J, Yao C, Che R, Zhang F, Zhao, D. Successive Layer-by-Layer Strategy for Multi-Shell Epitaxial Growth: Shell Thickness and Doping Position Dependence in Upconverting Optical Properties.Chem. Mater. 2013; 25:106−112.
